# Clinical implications of systemic and local immune responses in human angiosarcoma

**DOI:** 10.1038/s41698-021-00150-x

**Published:** 2021-02-12

**Authors:** Jason Yongsheng Chan, Grace Fangmin Tan, Joe Yeong, Chee Wee Ong, Dave Yong Xiang Ng, Elizabeth Lee, Joanna Koh, Cedric Chuan-Young Ng, Jing Yi Lee, Wei Liu, Ru Xin Wong, Chin-Ann Johnny Ong, Mohamad Farid, Bin Tean Teh, Khee Chee Soo

**Affiliations:** 1 Cancer Science Institute of Singapore, National University of Singapore, Singapore, Singapore; 2 Division of Medical Oncology, National Cancer Centre Singapore, Singapore, Singapore; 3 Oncology Academic Clinical Program, Duke-NUS Medical School, Singapore, Singapore; 4 Department of Anatomical Pathology, Singapore General Hospital, Singapore, Singapore; 5 Institute of Molecular and Cell Biology, Singapore, Singapore; 6 Integrated Genomics Platform, National Cancer Centre Singapore, Singapore, Singapore; 7 Laboratory of Cancer Epigenome, Division of Medical Sciences, National Cancer Centre Singapore, Singapore, Singapore; 8 Division of Radiation Oncology, National Cancer Centre Singapore, Singapore, Singapore; 9 Department of Sarcoma, Peritoneal and Rare Tumours (SPRinT), Division of Surgery and Surgical Oncology, National Cancer Centre Singapore, Singapore, Singapore; 10 Division of Surgery and Surgical Oncology, Singapore General Hospital, Singapore, Singapore; 11 Program in Cancer and Stem Cell Biology, Duke-NUS Medical School, Singapore, Singapore; 12 Division of Cellular and Molecular Research, National Cancer Centre Singapore, Singapore, Singapore

**Keywords:** Sarcoma, Predictive markers

## Abstract

Angiosarcomas are a rare subtype of soft-tissue sarcomas which exhibit aggressive clinical phenotypes with limited treatment options and poor outcomes. In this study, we investigated the clinical relevance of the peripheral blood neutrophil-to-lymphocyte ratio (NLR) as a marker of systemic immune response, as well as its correlation with intra-tumoral immune profiles in a subgroup of cases (*n* = 35) using the NanoString PanCancer IO360 panel and multiplex immunohistochemistry. In the overall cohort (*n* = 150), angiosarcomas of the head and neck (AS-HN) comprised most cases (58.7%) and median overall survival (OS) was 1.1 year. NLR, classified as high in 78 of 112 (70%) evaluable patients, was independently correlated with worse OS (HR 1.84, 95%CI 1.18–2.87, *p* = 0.0073). Peripheral blood NLR was positively correlated with intra-tumoral NLR (tNLR) (Spearman’s rho 0.450, *p* = 0.0067). Visualization of tumor-infiltrating immune cells confirmed that tNLR scores correlated directly with both neutrophil (CD15^+^ cells, rho 0.398, *p* = 0.0198) and macrophage (CD68^+^ cells, rho 0.515, *p* = 0.0018) cell counts. Interestingly, tNLR correlated positively with oncogenic pathway scores including angiogenesis, matrix remodeling and metastasis, and cytokine and chemokine signaling, as well as myeloid compartment scores (all *p* < 0.001). In patients with documented response assessment to first-line chemotherapy, these pathway scores were all significantly higher in non-responders (47%) compared to responders. In conclusion, systemic and local immune responses may inform chemotherapy response and clinical outcomes in angiosarcomas.

## Introduction

Angiosarcomas represent a rare subtype of soft-tissue sarcomas and are aggressive malignant mesenchymal tumors of endothelial cell origin. In terms of clinical presentation and behavior, these tumors often exhibit significant heterogeneity, and can develop in various anatomical structures, including the head and neck region, breast, viscera, trunk, and extremities^1,2^. Notwithstanding their rarity, several predisposing risk factors have been previously described. Angiosarcomas often arise in distinct clinical settings, either de novo (primary), following exposure to radiation, exogenous toxins, or in the presence of chronic lymphedema, foreign bodies, and arteriovenous fistulas (secondary)^2^. In the Asia-Pacific region, the epidemiological distribution of sarcomas remains poorly defined. In a previous observational study across five Asian countries, the most common histological subtypes include leiomyosarcoma, undifferentiated pleomorphic sarcoma, liposarcoma, synovial sarcoma, and angiosarcoma^3^. Interestingly, the proportion of angiosarcomas was larger as compared to that reported in Western studies (7.5% vs. 1–2%, respectively). More recently, we reported that angiosarcomas were amongst the commonest sarcoma subtypes in the elderly population, accounting for one-fifth of all cases diagnosed^4^. Furthermore, angiosarcomas of the head and neck (AS-HN) have been shown to be more prevalent in Asian populations, as compared with Western patients, raising the possibility of unique genetic or environmental factors influencing its pathogenesis^5^. In a large patient-partnered US-Canadian research initiative (The Angiosarcoma Project)^6^, 80% of the AS-HN cases harbored evidence of ultraviolet mutagenesis, whereas our recent study on the genomic landscape of angiosarcomas in an Asian population reported only 50% of cutaneous AS-HN with ultraviolet signatures^5^.

Regardless of etiology or anatomical origin, angiosarcomas are characterized by a challenging clinical course with limited treatment options and dismal prognosis. Contemporary treatment of localized angiosarcoma often involves surgical resection with wide margins, with or without adjuvant radiotherapy. In the metastatic setting, chemotherapeutic agents including paclitaxel, doxorubicin, or targeted agents are typically administered, although this is often met with rapid drug resistance^7^. More recently, responses to checkpoint immunotherapy in chemorefractory angiosarcomas have generated interest to better characterize their immune microenvironment^8–11^, and its combination with chemotherapy is currently under investigation in prospective clinical trials^12^.

As a hallmark of cancer, the inflammatory response plays an influential role throughout the course of tumor development^13,14^, contributing to immune escape, genetic instability, and reduced therapeutic response^15^. We and others have shown that an increased circulating peripheral blood neutrophil-to-lymphocyte ratio (NLR) was associated with adverse biological features and clinical outcome in soft-tissue sarcomas^16^. Furthermore, the NLR has been suggested as a potential predictive biomarker of immunotherapy response in sarcomas^17^. Specifically in angiosarcomas, we recently described the existence of unique genomic subtypes, including a distinct subset of AS-HN enriched for immune-related signaling and immune cells^5^. A deeper understanding of how the NLR relates to the intra-tumoral immune milieu and oncogenic signaling pathways may allow the discovery of novel therapeutic approaches in angiosarcoma.

In this study, we performed a retrospective analysis of angiosarcoma patients in an Asian tertiary cancer center, reviewing their clinical features, treatment outcomes and prognosis. Simultaneously, the systemic immune responses as well as intra-tumoral immune-oncological signaling pathways are examined and their clinical relevance explored.

## Results

### Patient demographics and survival analysis of overall cohort

The median age at diagnosis was 67 years (range, 26–104 years). Ninety-four (62.7%) were male and 56 (37.3%) were female. The majority of the patients were ethnic Chinese (82.7%). Most cases originated from the head and neck (58.7%). Other sites included the breast (8.7%), limb/trunk (8.7%), and visceral organs (25%). Eighteen patients (12.0%) had reported specific risk factors for angiosarcoma, including prior irradiation at the site of tumor origin (*n* = 8), chronic lymphedema (*n* = 3), and disease originating from a thrombosed arteriovenous fistula (*n* = 3). Out of 90 patients (60.0%) with localized disease, only 48 patients underwent surgical resection with curative intent. The rest were either considered surgically unresectable (*n* = 35) or did not undergo surgery for medical/other reasons (*n* = 7). Four patients were ECOG 4 at diagnosis and received supportive care alone. Patient characteristics are summarized in Table 1.

**Table 1:** Clinical features of patients with angiosarcoma included in the cohort.

Clinical characteristics	*n*
Total number of patients	150 (100%)
Sex	
Male	94 (62.7%)
Female	56 (37.3%)
Age at diagnosis (years)	
Median (range)	67 (26–104)
> 65	83 (55.3%)
≤ 65	67 (44.7%)
Ethnicity	
Chinese	124 (82.7%)
Other	26 (17.3%)
Performance status (ECOG score)	
0	70 (49.0%)
1	52 (36.4%)
2	11 (7.7%)
3	6 (4.2%)
4	4 (2.8%)
Cardiovascular risk factors^†^	
Present	90 (60.0%)
Absent	60 (40.0%)
Primary tumor site	
Head & neck	88 (58.7%)
Scalp	72 (48%)
Non-scalp	16 (10.7%)
Breast	13 (8.7%)
Limb/trunk	13 (8.7%)
Other^‡^	36 (24%)
Etiology	
Primary	132 (88.0%)
Secondary	18 (12.0%)
	Radiation (*n* = 8)
	Chronic lymphedema (*n* = 3)
	Thrombosed arteriovenous fistula (*n* = 3)
	Organ transplant (*n* = 1)
	Neurofibromatosis Type 1 (*n* = 1)
	Li-Fraumeni syndrome (*n* = 1)
	Xeroderma pigmentosum (*n* = 1)
Epithelioid component	
Present	59 (51.3%)
Absent	56 (48.7%)
Distant metastasis at diagnosis	
Present	60 (40.0%)
Absent	90 (60.0%)
Curative surgery	48 (32.0%)
Other^*^	42 (28.0%)

^†^Hypertension, hyperlipidemia, diabetes mellitus, ischemic heart disease, cerebrovascular disease.

^‡^Sites include liver (*n* = 13), spleen/heart/peritoneum (*n* = 3 each), pleura/bone/small bowel/prostate (*n* = 2 each), brachial plexus/kidney/ovary/vagina (*n* = 1 each), unknown (*n* = 2).

^*^Surgically unresectable (*n* = 35), medical/other reasons (*n* = 7).

Data unavailable for ECOG score (*n* = 7), histomorphology (*n* = 35).

**Fig. 1:**
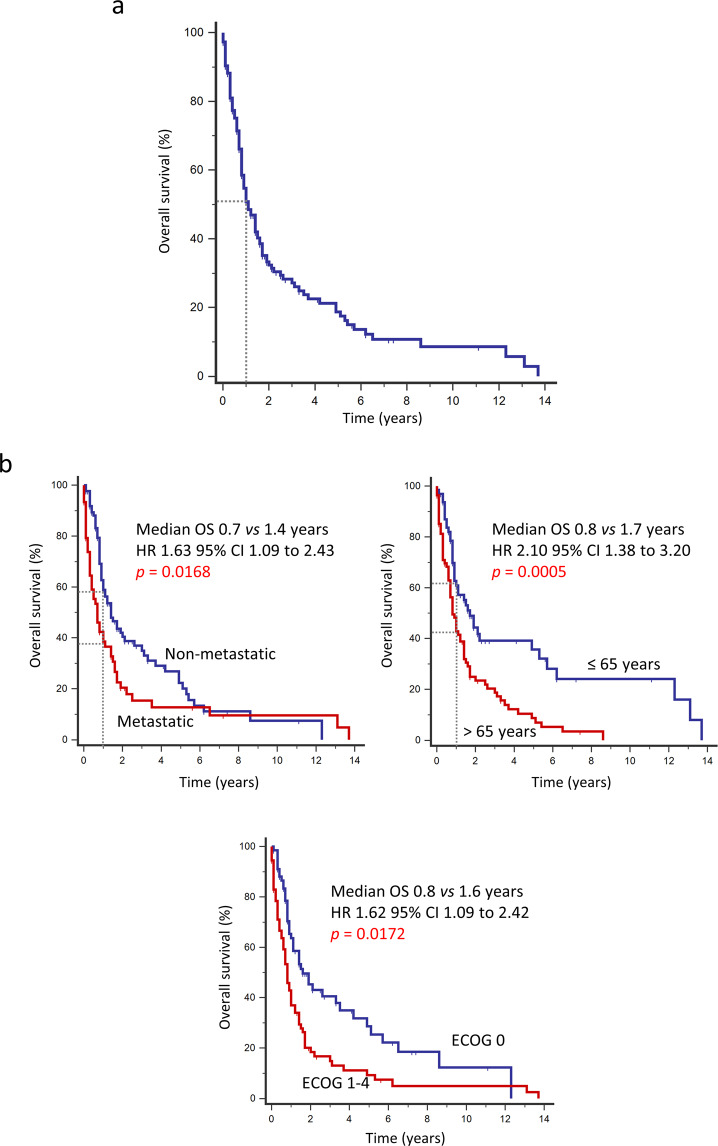
Overall survival outcomes and prognostic factors in angiosarcoma. **a** In the overall cohort (*n* = 150), 50.8% of the patients were alive at 1 year. Median OS was 1.1 year. **b** Independent predictors of OS at the time of diagnosis include the presence of distant metastasis, age >65 years, and poor performance status (ECOG 1–4).

AS-HN were more common in men (*p* = 0.0192), elderly patients >65 years old (*p* < 0.0001), and associated with cardiovascular co-morbidities (*p* < 0.0001). Most cases were primary angiosarcomas with no reported risk factors (*p* < 0.0001), of cutaneous origin (*p* < 0.0001) and were less commonly associated with distant metastasis at the time of diagnosis (*p* = 0.0365). Primary tumor site was not associated with ethnicity (Table 2). At the time of data analysis, 111 patients (74.0%) had died. In the overall cohort, only 50.8% of patients were alive at 1 year. Median overall survival (OS) was 1.1 year (Fig. 1a). Independent predictors of OS at the time of diagnosis include the presence of distant metastasis (HR 1.63, 95% CI 1.09–2.43, *p* = 0.0168), age >65 years (HR 2.10, 95% CI 1.38–3.20, *p* = 0.0005), and performance status (ECOG 1–4) (HR 1.62, 95% CI 1.09–2.42, *p* = 0.0172) (Fig. 1b). These factors were similarly predictive for disease-specific survival (DSS) (Tables 3–4). Expectedly, patients with localized angiosarcoma who did not undergo curative surgery did poorer in terms of OS (HR 1.64, 95% CI 0.97–2.78, *p* = 0.0641) and DSS (HR 2.01, 95% CI 1.15–3.50, *p* = 0.0140) (Supplementary Fig. 1).

**Table 2:** Clinical features of head & neck angiosarcoma (AS-HN) compared to other sites.

Characteristic (*n*)	Tumor site		*p*
	AS-HN	Other	
Total (150)	88 (58.7%)	62 (41.3%)	–
Sex			0.0192
Male (94)	62 (66.0%)	32 (34.0%)	
Female (56)	26 (46.4%)	30 (53.6%)	
Age at diagnosis (years)			<0.0001
>65 (83)	67 (80.7%)	16 (19.3%)	
≤65 (67)	21 (31.3%)	46 (68.7%)	
Ethnicity			0.156
Chinese (124)	76 (61.3%)	48 (38.7%)	
Other (26)	12 (46.2%)	14 (53.8%)	
Performance status (ECOG score)			0.118
0 (70)	36 (51.4%)	34 (48.6%)	
1–4 (73)	47 (64.4%)	26 (35.6%)	
Cardiovascular co-morbidities^†^			<0.0001
Present (90)	65 (72.2%)	25 (27.8%)	
Absent (60)	23 (38.3%)	37 (61.7%)	
Etiology			<0.0001
Primary (132)	87 (65.9%)	45 (34.1%)	
Secondary (18)	1 (5.6%)	17 (94.4%)	
Cutaneous origin			<0.0001
Yes (90)	83 (92.2%)	7 (7.8%)	
No (60)	5 (8.3%)	55 (91.7%)	
Epithelioid component			0.393
Present (59)	29 (49.2%)	30 (50.8%)	
Absent (56)	32 (57.1%)	24 (42.9%)	
Distant metastasis at diagnosis			0.0365
Present (60)	29 (48.3%)	31 (51.7%)	
Absent (90)	59 (65.6%)	31 (34.4%)	

^†^Includes hypertension, hyperlipidemia, diabetes mellitus, ischemic heart disease, cerebrovascular disease.

Data unavailable for ECOG score (*n* = 7), histomorphology (*n* = 35).

**Table 3:** Univariate survival analysis of the entire study cohort.

	Overall survival		Disease-specific survival	
Characteristic	HR (95% CI)	*p*	HR (95% CI)	*p*
Sex (male vs female)	1.11 (0.75–1.64)	0.616	1.06 (0.71–1.60)	0.773
Age at diagnosis (>65 vs ≤65 years)	2.10 (1.42–3.11)	0.0002	2.03 (1.36–3.04)	0.0006
Ethnicity (Chinese vs other)	1.30 (0.77–2.19)	0.330	1.20 (0.70–2.07)	0.507
Cardiovascular co-morbidities (present vs absent)	1.27 (0.86–1.88)	0.233	1.28 (0.85–1.93)	0.233
Performance status (ECOG 1–4 vs 0)	2.03 (1.36–3.02)	0.0005	2.2920 (1.52–3.46)	0.0001
Primary tumor site (AS-HN vs other)	1.00 (0.67–1.48)	0.988	0.93 (0.61–1.39)	0.710
Etiology (secondary vs primary)	1.09 (0.60–1.98)	0.778	1.13 (0.61–2.12)	0.694
Epithelioid component (present vs absent)	1.43 (0.92–2.23)	0.114	1.28 (0.81–2.03)	0.285
Distant metastasis at diagnosis (present vs absent)	1.71 (1.13–2.59)	0.0112	2.06 (1.34–3.17)	0.0010

Abbreviations: AS-HN, angiosarcoma of the head and neck.

**Table 4:** Multivariate survival analysis of the entire study cohort.

	Overall survival		Disease-specific survival	
Characteristic	HR (95% CI)	*p*	HR (95% CI)	*p*
Age at diagnosis (>65 vs ≤65 years)	2.10 (1.38–3.20)	0.0005	2.05 (1.33–3.15)	0.0012
Performance status (ECOG 1–4 vs 0)	1.62 (1.09–2.42)	0.0172	1.80 (1.19–2.73)	0.0058
Distant metastasis at diagnosis (present vs absent)	1.63 (1.09–2.43)	0.0168	1.89 (1.25–2.85)	0.0024

### Localized angiosarcoma treated with curative surgery

Amongst patients with localized angiosarcoma who underwent curative resection (*n* = 48), half (50%) the cases were AS-HN. Three patients who underwent radical lymph node dissection for regional lymph node involvement were included in this analysis. The median tumor size was 5 cm (range: 1–23 cm). Tumors were resected with R0 margins in 35 cases (76.1%) and with R1 margins in 11 cases (23.9%). Post-operative radiation therapy was administered to 22 patients (45.8%) (Supplementary Table 1). Reasons for not undergoing radiation therapy include patient preference (*n* = 5), anatomical reasons (*n* = 5), wound issues (*n* = 4), prior radiation therapy at tumor site (*n* = 2), xeroderma pigmentosum (*n* = 1), and unknown (*n* = 9). At the time of analysis, 28 patients (58.3%) had died. Median OS was 1.9 years and median RFS was 0.7 years. Five-year survival estimates were 32.8% for OS and 17.4% for RFS. A total of 30 patients had presented with disease relapse at a median of 5 months (range, 1.1–29.5 months) following initial surgery. The patterns of relapse include distant (*n* = 12), locoregional only (*n* = 8), and locoregional followed by subsequent distant metastasis (*n* = 10). Out of those who had died, the majority (*n* = 19, 86.4%) had relapsed with distant metastasis. Six patients died of causes probably unrelated to angiosarcoma, including infections (*n* = 3), metastatic nasopharyngeal cancer (*n* = 1), and unknown (*n* = 2). Previous studies have demonstrated that positive resection margins were strongly associated with OS^18,19^. Consistent with these findings, our results showed that amongst several clinical variables examined, only the presence of microscopic positive margins was independently correlated with worse OS (HR 2.60, 95% CI 1.06–6.36, *p* = 0.0367) and RFS (HR 3.25, 95% CI 1.43–7.38, *p* = 0.0047) (Supplementary Fig. 2 and Supplementary Tables 2 and 3).

### Patterns of metastasis and prognostic outcomes of metastatic angiosarcoma

**Fig. 2:**
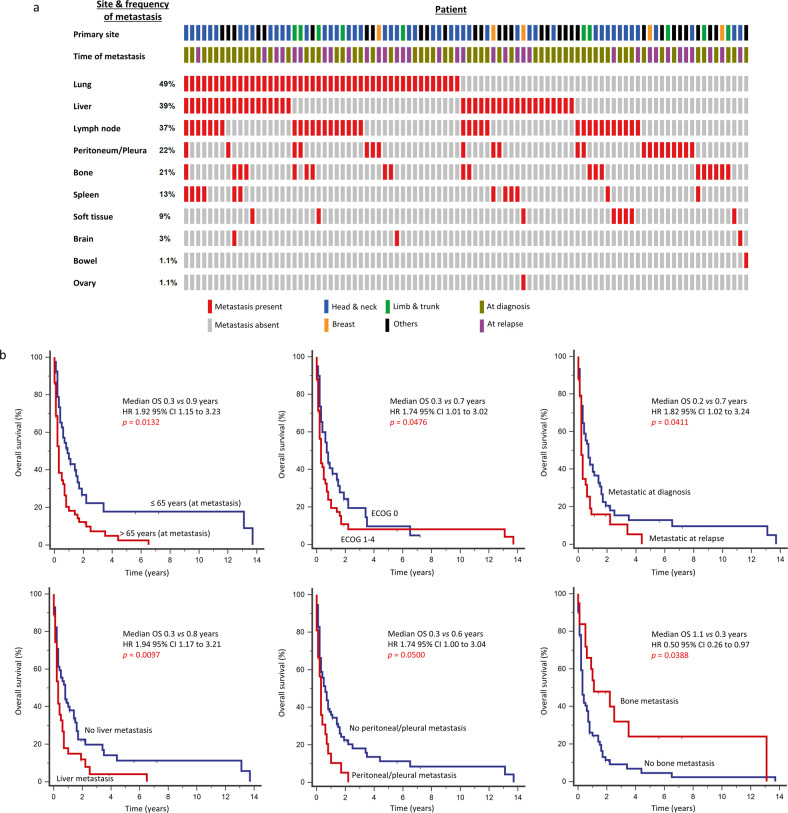
Patterns of dissemination and survival outcomes of metastatic angiosarcoma. **a** The commonest sites of metastases include the lungs (49%), liver (39%), and lymph nodes (37%). **b** Independent predictors of poor OS at the time of metastasis include age >65 years, poor performance status (ECOG 1–4), metastasis at relapse, and the presence of liver or peritoneal/pleural metastases. The presence of bone metastasis was associated with better OS.

A total of 94 patients were diagnosed with distant metastatic disease, either at diagnosis (*n* = 60; 63.8%), or at relapse (*n* = 34; 36.2%). The lung was the most frequent site involved (49%), followed by the liver (39%) and lymph node (37%). Metastases to the brain and bowel were rare (3% and 1.1%, respectively) (Fig. 2a). AS-HN were more likely to develop metastases to the lungs (*p* = 0.0237) and lymph nodes (*p* = 0.0093), whilst peritoneal and pleural metastases were more common in non-AS-HN (*p* = 0.0011) (Supplementary Table 4).

Seventy-nine patients (84.0%) had died at the time of analysis. In a multivariable model, we demonstrated that in patients with metastatic angiosarcoma, age >65 years (HR 1.92, 95% CI 1.15–3.23, *p* = 0.0132) and poor performance status (HR 1.74, 95% CI 1.01–3.02, *p* = 0.0476) were independent indicators of poor OS. Other independent poor prognostic factors include the development of metastasis upon relapse rather than at initial diagnosis (HR 1.82, 95% CI 1.02–3.24, *p* = 0.0411), the presence of liver metastases (HR 1.94, 95% CI 1.17–3.21, *p* = 0.0097), and the presence of peritoneal/pleural metastases (HR 1.74, 95% CI 1.00–3.04, *p* = 0.0500). Conversely, bone metastases (HR 0.50, 95% CI 0.26–0.97, *p* = 0.0388) and receipt of palliative chemotherapy (HR 0.42, 95% CI 0.25–0.70, *p* = 0.0011) were independent predictors of better OS (Fig. 2b and Supplementary Tables 5 and 6).

### Response to palliative chemotherapy

A total of 71 patients (47.3%) received palliative chemotherapy. Most had undergone only a single line of chemotherapy (57.7%), and the rest had two (16.9%) or more (25.4%) lines. Sixty patients had best clinical responses to first-line chemotherapy documented, including 47 patients with available computed tomography (CT) images for further analysis by RECIST response criteria^20^. For the remaining patients (*n* = 13), responses to chemotherapy were evaluated clinically by the individual managing oncologist, either because of obvious clinical progression (*n* = 5) or non-measurable lesions on radiological imaging (*n* = 8). In the overall cohort (*n* = 60), partial response (PR) was achieved in 32 patients (53.3%) and stable disease (SD) in 14 patients (23.3%), resulting in a clinical benefit rate of 76.7% (Table 5). Patients with non-measurable disease on imaging were assessed as PR (bone, *n* = 2; skin, *n* = 3) or SD (skin, *n* = 3).

**Table 5:** Palliative chemotherapy received by patients with angiosarcoma.

Clinical characteristics	*n*				
Received palliative chemotherapy					
Yes	71 (47.3%)				
No	76 (50.7%)				
Unknown	3 (2.0%)				
Lines of palliative chemotherapy					
1	41 (57.7%)				
2	12 (16.9%)				
≥3	18 (25.4%)				
First-line palliative chemotherapy					
Paclitaxel	53 (74.6%)				
Doxorubicin-based^†^	12 (16.9%)				
Other^‡^	6 (8.5%)				
Second-line palliative chemotherapy					
Paclitaxel	6 (20.0%)				
Doxorubicin-based^††^	18 (60.0%)				
Other^‡‡^	6 (20.0%)				
Response to first-line chemotherapy by radiological and/or clinical assessment^§^	Overall (*n* = 60)	Paclitaxel			Doxorubicin-based^*^ (*n* = 11)
		AS-HN (*n* = 31)	Other (*n* = 14)	Any site (*n* = 45)	
Progressive disease	14 (23.3%)	3 (9.7%)	2 (14.3%)	5 (11.1%)	7 (63.6%)
Stable disease	14 (23.3%)	5 (16.1%)	5 (35.7%)	10 (22.2%)	2 (18.2%)
Partial response	32 (53.3%)	23 (74.2%)	7 (50.0%)	30 (66.7%)	2 (18.2%)
Response to first-line chemotherapy by radiological assessment only^¶^	Overall (*n* = 47)	Paclitaxel			Doxorubicin-based (*n* = 8)
		AS-HN (*n* = 27)	Other (*n* = 11)	Any site (*n* = 38)	
Progressive disease	9 (19.1%)	3 (11.1%)	2 (18.2%)	5 (13.2%)	4 (50.0%)
Stable disease	11 (23.4%)	3 (11.1%)	5 (45.5%)	8 (21.1%)	2 (25.0%)
Partial response	27 (57.4%)	21 (77.8%)	4 (36.4%)	25 (65.8%)	2 (25.0%)

^†^Doxorubicin plus ifosfamide (*n* = 3), doxorubicin alone (*n* = 2) and liposomal doxorubicin (*n* = 7).

^‡^Ifosfamide (*n* = 1), thalidomide (*n* = 2), sunitinib (*n* = 1) and unknown (*n* = 2).

^††^Liposomal doxorubicin (*n* = 17) and doxorubicin alone (*n* = 1).

^‡‡^Gemcitabine plus cisplatin (*n* = 1), thalidomide plus cyclophosphamide (*n* = 1), bevacizumab (*n* = 3) and unknown (*n* = 1).

^*^Only 1 AS-HN primary with progressive disease.

^§^All patients with response documentation, including by clinical evaluation only (*n* = 13).

^¶^Includes only patients with response documentation as assessed by radiological imaging.

Abbreviations: AS-HN, angiosarcoma of the head and neck.

**Fig. 3:**
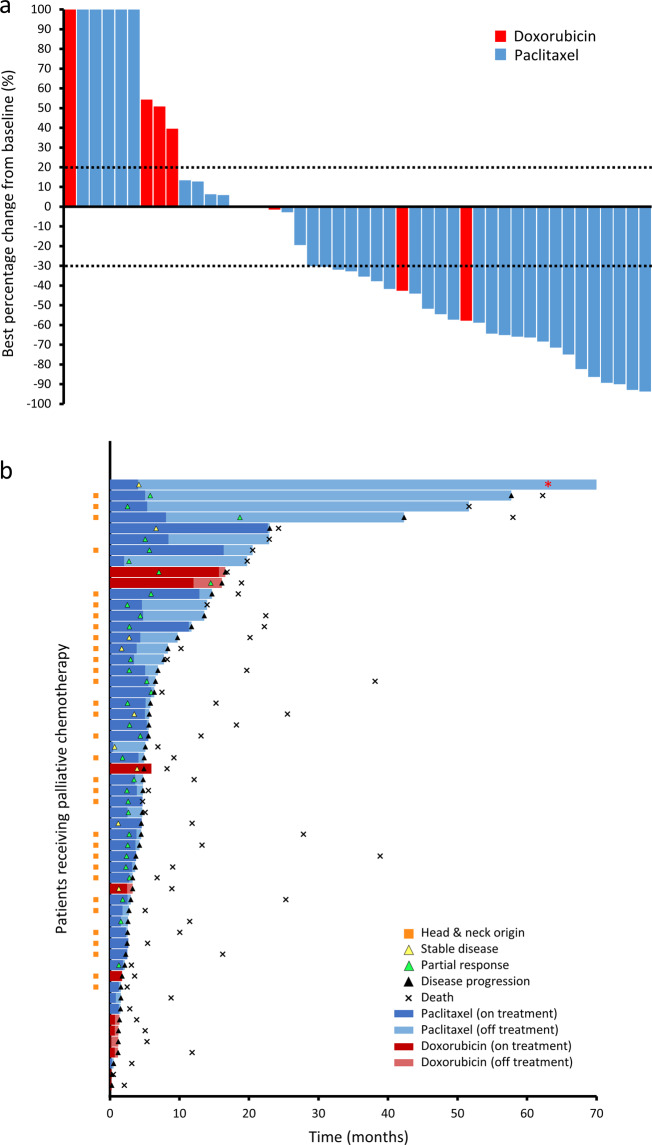
Response to first-line chemotherapy in angiosarcoma. **a** First-line chemotherapy resulted in partial responses in 57.4% and stable disease in a further 23.4% of patients. **b** The median time-to-progression from first-line chemotherapy was 4.8 months (95% CI 3.7–6.4 months). Red asterisk: still alive and disease-free at 148 months.

The optimal first-line chemotherapy option for angiosarcoma remains controversial. In our cohort, weekly paclitaxel (typically 80 mg/m^2^/day on days 1, 8, 15, repeating every 28 days) was the most commonly used first-line chemotherapeutic regimen (74.6%), followed by doxorubicin-based regimens (16.9%), and others (8.5%). Significantly higher response rates were observed using paclitaxel (30 out of 45 patients, 66.7%) compared to doxorubicin-based regimens (2 out of 11 patients, 18.2%) (*p* = 0.00566, Fisher’s exact test). The response rates to paclitaxel were similar in AS-HN (74.2%) as compared to other sites (50.0%) (*p* = 0.115). Similar response rates were observed in the subset of patients evaluated by CT imaging using RECIST v1.1 criteria. The treatment responses to first-line chemotherapy and their clinical courses are summarized in a waterfall plot and swimmer plot, respectively (Fig. 3). The median time to progression (TTP) was 4.8 months (95% CI 3.7–6.4) in the overall cohort. TTP was significantly longer for paclitaxel (5.5 months, 95% CI 4.5–6.9) compared to doxorubicin-based regimens (1.4 months, 95% CI 0.3–16.6) (HR 2.91, 95% CI 1.16–7.32, *p* = 0.023). Consistent with the observation that palliative chemotherapy improves survival outcomes, responses to first-line palliative chemotherapy were correlated with improved OS (*p* = 0.0013) (Supplementary Fig. 3).

Thirty patients received second-line chemotherapy, including paclitaxel (*n* = 6, all following doxorubicin-based regimens), doxorubicin (*n* = 12, all following paclitaxel), and others (bevacizumab, *n* = 3; cyclophosphamide plus thalidomide, *n* = 1; gemcitabine plus cisplatin, *n* = 1; unknown, *n* = 1). PRs were observed in only 4 patients (1 paclitaxel, 3 doxorubicin), and median TTP was 2.1 months.

### Peripheral blood neutrophil-to-lymphocyte ratios

We and others have previously shown that the NLR has prognostic significance in various cancers, including soft-tissue sarcomas^16,21^. To further substantiate this observation, we examined a subcohort of patients with available peripheral blood counts obtained at the time of diagnosis and investigated the prognostic role of the NLR. Across the entire cohort, the values for NLR ranged from 0.8 to 68.6 (median: 3.3), and were classified as NLR-high (>2.5) in 78 patients (69.6%) (Supplementary Table 7). NLR and absolute neutrophil counts were positively correlated (Spearman’s rho 0.720, 95% CI 0.617–0.799, *p* < 0.0001), while an opposite correlation with absolute lymphocyte counts was demonstrated (Spearman’s rho −0.683, 95% CI −0.771 to −0.570, *p* < 0.0001) (Supplementary Fig. 4).

**Fig. 4:**
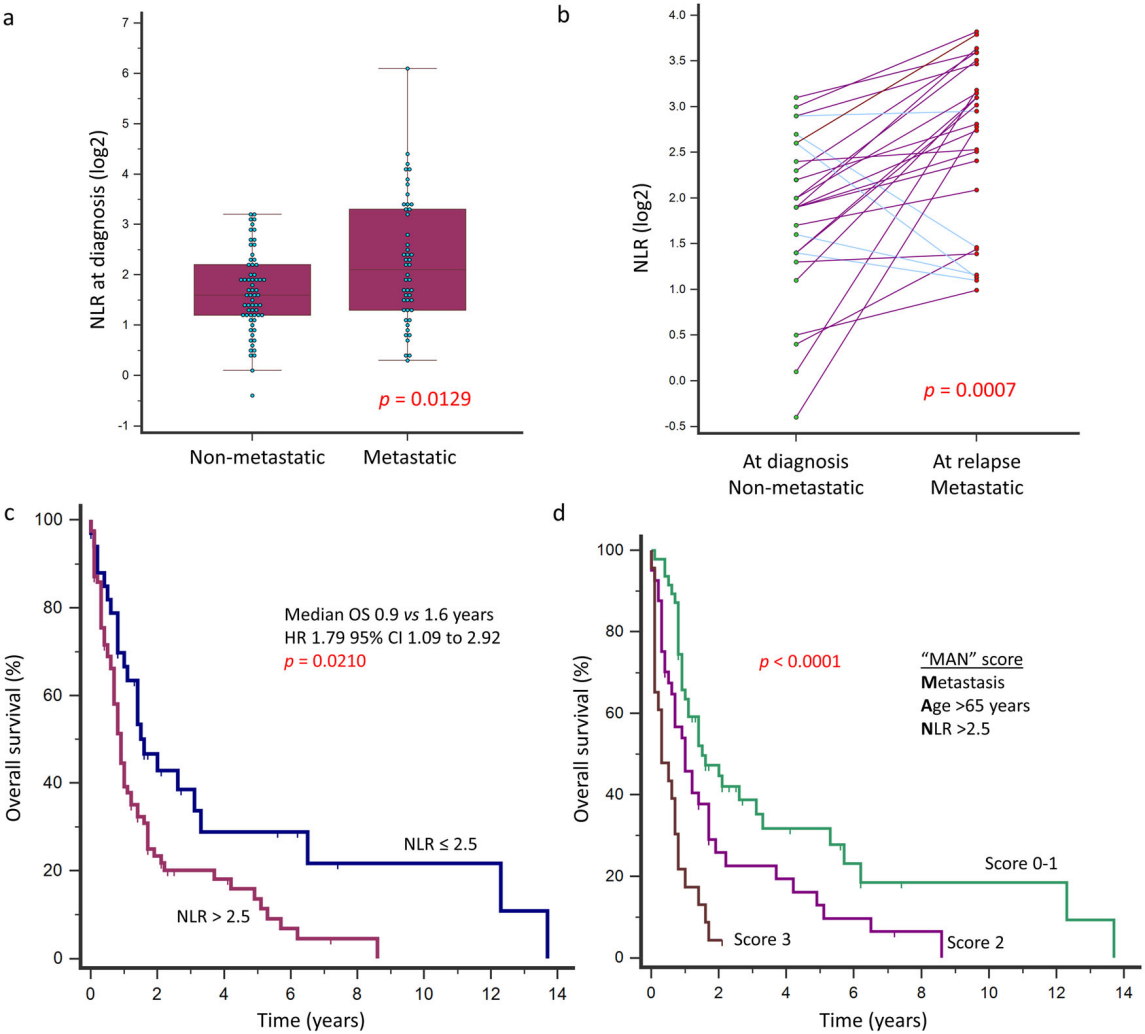
Peripheral blood NLR and survival outcomes in angiosarcoma. **a** Patients with distant metastasis at diagnosis had higher NLR (*p* = 0.0129, Mann-Whitney *U* test) (Boxplot elements: center line, median; bounds of box, lower and upper quartiles; whiskers, minimum and maximum values.). **b** Progression of localized disease at diagnosis to metastatic relapse within the same patients was associated with an increase in NLR (*p* = 0.0007, Wilcoxon signed-rank test). **c** High NLR > 2.5 was independently associated with worse OS. **d** Patients were risk-stratified based on the “MAN” prognostic score into low (0–1), intermediate (2), and high (3) risk subgroups, which were associated with 1-year OS of 63.5%, 45.9%, and 17.4%, respectively.

In patients with distant metastasis at diagnosis compared to those without, NLR was significantly higher (median: 4.3 vs 3.0, *p* = 0.0129) (Fig. 4a). This was associated with both higher neutrophil counts (median: 5.1 vs 3.7, *p* = 0.3349) as well as lower lymphocyte counts (median: 1.7 vs 1.3, *p* = 0.0194). In patients without metastasis, 28 (25%) eventually developed distant metastasis following a median of 11.4 months. Peripheral blood neutrophil and lymphocyte counts before treatment at the time of diagnosis and upon metastatic relapse were available for 27 patients. An interval increase in NLR was demonstrated in these patients on progression of localized disease at diagnosis to metastatic relapse (median: 3.8 vs 7.0, *p* = 0.0007) (Fig. 4b). This finding was correlated with an interval decrease in lymphocyte counts (median: 1.2 vs 0.9, *p* = 0.0795) as well as a rise in neutrophil counts (median: 4.6 vs 6.3, *p* = 0.0411). Like our previously reported results^16^, NLR-high was significantly associated with worse OS (HR 1.84, 95% CI 1.18–2.87, *p* = 0.0073), and remained as an independent predictor for OS in a multivariable model along with age at diagnosis >65 years and the presence of distant metastasis at diagnosis (Fig. 4c and Supplementary Tables 8 and 9). Using these three variables (termed the “MAN” prognostic score), patients could be risk-stratified into low (0–1), intermediate (2), and high (3) risk subgroups. These represented 42.9%, 36.6%, and 20.5% of the patients, and were associated with 1-year OS of 63.5%, 45.9%, and 17.4%, respectively (Fig. 4d).

### Tumor immune-oncogenic signaling and chemotherapeutic resistance

**Fig. 5:**
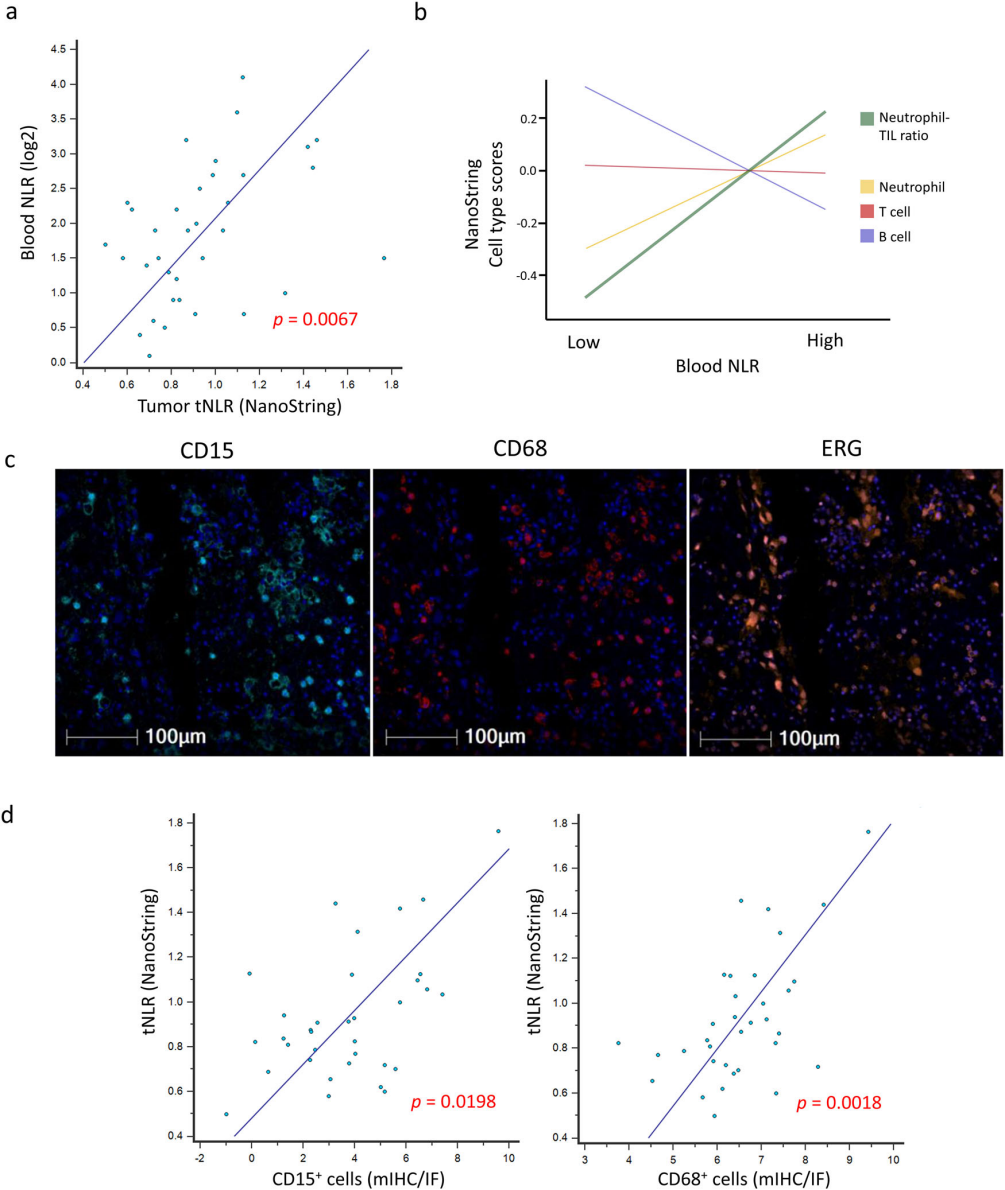
Correlation of peripheral blood NLR with tNLR. **a, b** Peripheral blood NLR showed a positive correlation with intra-tumoral neutrophil-to-lymphocyte (tNLR) as derived from NanoString transcriptomic profiling (Spearman’s rho = 0.450, *p* = 0.0067), and correlated appropriately with corresponding cell-type scores. **c** Tumor-infiltrating immune cells were visualized via Multiplex Immunohistochemistry/Immunofluorescence (mIHC/IF). The proportion of neutrophils (CD15^+^), macrophages (CD68^+^), cytotoxic T-cells (CD8^+^), and regulatory T-cells (FOXP3^+^) relative to tumor cells (ERG^+^) were obtained and correlated with tNLR scores. **d** The tNLR scores correlated directly with CD15^+^ cells (Spearman’s rho 0.398, *p* = 0.0198) and CD68^+^ cells (Spearman’s rho 0.515, *p* = 0.0018) on mIHC/IF.

**Fig. 6:**
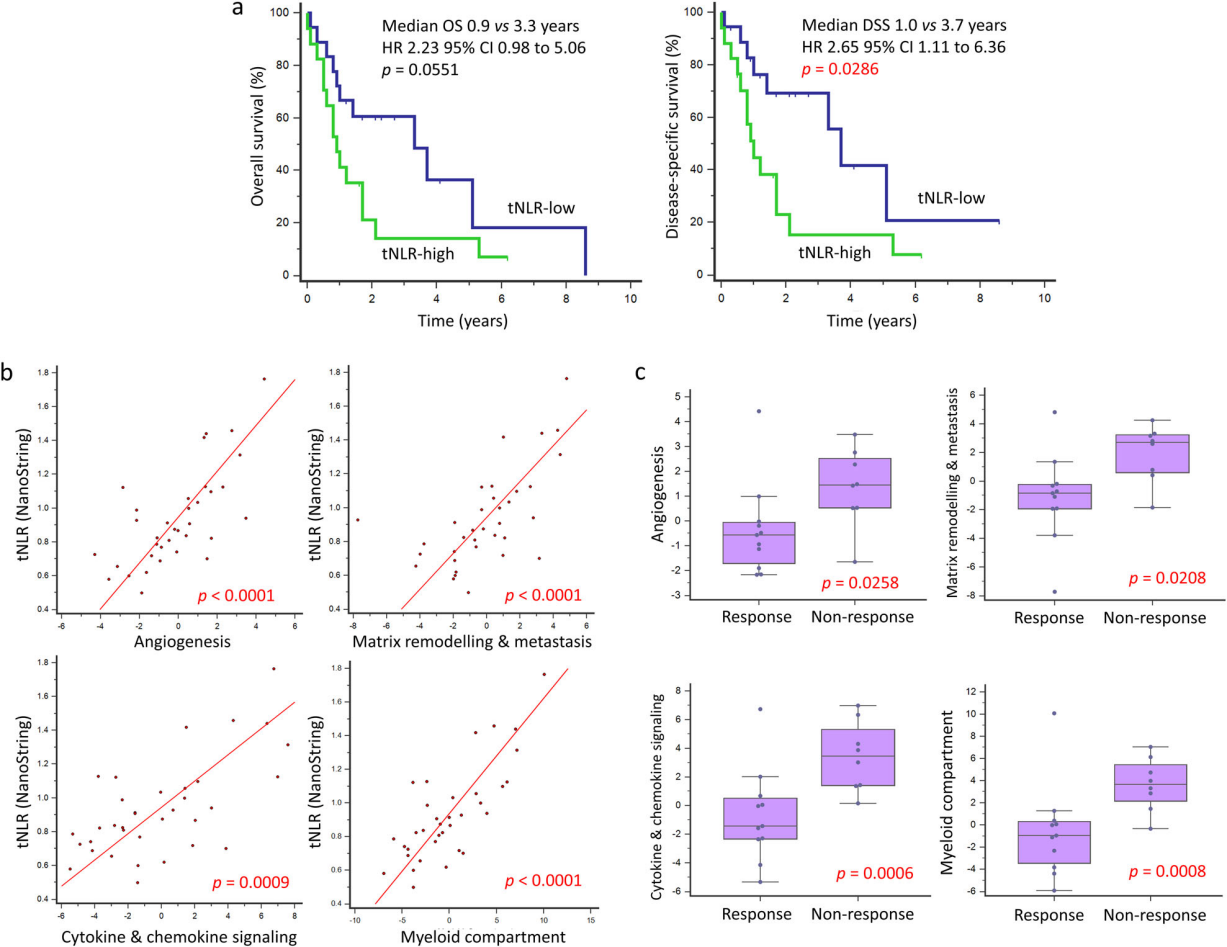
Correlation of tNLR with oncogenic signaling pathways and survival outcomes. **a** Similar to peripheral blood NLR, high tNLR scores conferred worse survival outcomes (OS: HR 2.23, 95%CI 0.98–5.06, *p* = 0.0551); DSS: HR 2.65, 95%CI 1.11–6.36, *p* = 0.0286). **b** tNLR correlated positively with oncogenic pathway scores including angiogenesis (rho = 0.664, *p* < 0.0001), matrix remodeling and metastasis (rho = 0.666, *p* < 0.0001), and cytokine and chemokine signaling (rho = 0.538, *p* = 0.0009), as well as myeloid compartment scores (rho = 0.689, *p* < 0.0001). **c** In patients with non-response to first-line palliative chemotherapy (SD or PD), scores for angiogenesis (*p* = 0.0258), matrix remodeling and metastasis (*p* = 0.0208), cytokine and chemokine signaling (*p* = 0.006), and myeloid compartment (*p* = 0.008) were all significantly higher compared to responders.

In an exploratory analysis, we studied the correlation of peripheral blood NLR with tumor-infiltrating immune cell types and oncogenic pathways as inferred from bulk transcriptomes using the NanoString PanCancer IO360 panel. Peripheral blood NLR was positively correlated with tumor NLR (tNLR) (Spearman’s rho 0.450, *p* = 0.0067) (Fig. 5a). In corroboration, patients with high peripheral blood NLR ( > 2.5) demonstrated higher absolute tumor neutrophil and macrophage scores, as well as lower lymphocyte (B-cell and T-cell) scores (Fig. 5b). tNLR correlated positively with oncogenic pathway scores including angiogenesis (rho = 0.664, *p* < 0.0001), matrix remodeling and metastasis (rho = 0.666, *p* < 0.0001) and cytokine and chemokine signaling (rho = 0.538, *p* = 0.0009), as well as myeloid compartment scores (rho = 0.689, *p* < 0.0001) (Supplementary Table 10). In corroboration with this result, direct visualization of tumor-infiltrating immune cells confirmed that tNLR correlated directly with both neutrophil (CD15^+^ cells, Spearman’s rho 0.398, *p* = 0.0198) and macrophage (CD68^+^ cells, Spearman’s rho 0.515, *p* = 0.0018) cell counts (Fig. 5c-d and Supplementary Fig. 5). Similar to peripheral blood NLR, high tNLR scores conferred worse survival outcomes (OS: HR 2.23, 95% CI 0.98–5.06, *p* = 0.0551); DSS: HR 2.65, 95% CI 1.11–6.36, *p* = 0.0286) in this subcohort of patients with both non-metastatic (*n* = 26) and metastatic disease (*n* = 9) (Fig. 6a).

We next examined for any correlation between gene expression and chemotherapy response. A total of 118 genes were differentially expressed (*p* < 0.05) (Supplementary Table 11). A random forest classification-based Boruta algorithm was applied to the subset of significant genes (*p* < 0.05)^22^. The feature-selection process by Boruta identified 17 important genes (all upregulated in non-responders). Summation of their normalized log2 count data was able to predict chemotherapy response with a sensitivity of 90.9% and specificity of 100% at a cut-off of < 135 (Supplementary Fig. 6). In terms of pathway scores, scores for angiogenesis (*p* = 0.0259), matrix remodeling and metastasis (*p* = 0.0203), cytokine and chemokine signaling (*p* = 0.005), and myeloid compartment (*p* = 0.0068) were all significantly higher in non-responders compared to responders, in keeping with observations with tNLR (Fig. 6b and Supplementary Table 12). These findings will require confirmation in validation cohorts.

## Discussion

Our results demonstrate several unique features of angiosarcomas within this cohort of Asian patients. Confirming previous reports, most cases originated from the head and neck, in particular from the scalp and face^5,23^. This is in contrast to Western populations, in which AS-HN are less common, accounting for only 26.3% (US SEER database)^24^, 17% (Netherlands Cancer Registry)^25^ and 10.6% (French Sarcoma Group^1^ of angiosarcomas as per various studies. Regardless, the clinical presentation and outcomes are generally similar—AS-HN are more common in men and the elderly, and frequently associated with cardiovascular co-morbidities^26^. Despite its lower propensity for presenting with metastases de novo, the typical patient profile presents definite challenges in the optimal management of this debilitating disease. In our cohort, only a quarter of the patients managed to undergo curative surgery, and less than half received palliative chemotherapy. Unsurprisingly, the overall prognosis of patients with angiosarcoma is extremely dismal. The 1 year survival rate in our cohort of 50.8% is largely comparable to others (1 year OS: 55.2% [US SEER], 52.6% [Netherlands Cancer Registry])^24,25^. Poor prognostic factors identified including older age, poor performance status, and distant metastasis are also consistent with prior reports^2,8,24,25^. Amongst patients with metastatic angiosarcoma, the presence of liver, peritoneal, and pleural metastasis conferred worse survival outcomes. Interestingly, we observed that bone metastasis (present in one-fifth of these patients) was associated with improved survival—a finding in contrast to a previous retrospective analysis by the French Sarcoma Group, and will require further validation in future studies^8^.

In our cohort, weekly paclitaxel was the most commonly used palliative chemotherapy regimen. Early studies had suggested remarkable efficacy of paclitaxel in angiosarcomas of the scalp^27,28^, although responses were also subsequently observed in those arising from other primary sites^29^. In the ANGIOTAX phase II study, weekly paclitaxel induced PRs in 17% of patients (*n* = 30, majority of breast and visceral origins), including 36% who received prior anthracycline-based chemotherapy^30^. In the ANGIOTAX-PLUS phase II study evaluating bevacizumab in addition to paclitaxel, patients randomized to the paclitaxel arm (*n* = 24) achieved a PR rate of 45%^31^. In our cohort, PR to paclitaxel was 67% compared to 18% for doxorubicin-based regimens. In line with this result, previous studies have similarly suggested that paclitaxel induces superior response rates over doxorubicin-containing regimens (53% vs 30% in Italiano et al., 2012; 46% vs 31% in Penel et al., 2012), though this has been attributed to the higher sensitivity of cutaneous angiosarcomas to paclitaxel and imbalances in study groups^7,32,33^. Indeed, our own results showed that AS-HN (74%) were indeed more responsive to paclitaxel as compared to other sites (50%), an observation in line with that reported in a previous EORTC series (75% for scalp and 58% for other sites)^34^. In a pooled analysis of 108 angiosarcoma patients (90% non-cutaneous) treated with first-line anthracycline-based chemotherapy as part of prospective EORTC trials, the response rate was only 25%^35^. Taken together, although the choice of first-line palliative chemotherapy for angiosarcoma remains controversial, paclitaxel may present as a more palatable option given its high response rates and favorable toxicity profile for the typical elderly patient with AS-HN.

Recently, we have shown that systemic immune responses to the development of soft-tissue sarcoma varies according to biological phenotype and clinical behavior^16^. Specifically, we found that a high baseline peripheral blood NLR was associated with distant metastasis, higher tumor grade, larger tumor size, as well as greater tumor depth. Confirming our previous result, we showed that high NLR was significantly correlated with worse OS, and could be incorporated into a prognostic score for patient risk stratification. More interestingly, the intra-tumoral immune milieu (tNLR) mirrored the systemic response, and was associated with the upregulation of several immune-oncology pathways associated with primary resistance to chemotherapy, including angiogenesis, matrix remodeling and metastasis, cytokine and chemokine signaling, as well as the accumulation of neutrophils and macrophages. It may be hypothesized that the observed immune responses may be attributed in part to tumor-related signals that promote metastasis, and mechanistic studies are warranted to understand these observations in detail. Importantly, these findings may represent opportunities for the discovery of novel therapeutics in angiosarcoma.

Mutations in angiogenesis signaling genes such as *KDR*^36^, *PTPRB*, and *PLCG1*^37^, as well as the expression of angiogenic growth factors are known to occur in angiosarcomas^38^. Given that paclitaxel is a microtubule-targeting drug with anti-angiogenic properties^39,40^, altered angiogenic signaling leading to its resistance is not unexpected, yet attempts to further exploit this pathway for therapy have been largely unsuccessful. The addition of bevacizumab to weekly paclitaxel has been studied in a non-comparative open-label randomized phase II trial. The response rate to combination therapy was not only lower (29% vs 46%), these patients also experienced more frequent severe toxicities^31^. The evaluation of circulating pro/anti-angiogenic factors did not identify any subgroup that benefited from the addition of bevacizumab^41^. More recently, TRC105, an antibody against an essential angiogenic receptor known as endoglin, did not demonstrate significant activity when combined with pazopanib, a tyrosine kinase inhibitor with anti-angiogenic activity^42^. Angiosarcomas are enriched for myeloid cell types including neutrophils and macrophages (in particular tumor-promoting M2 macrophages)^5,43^. Tumor-associated myeloid cells have been shown to modulate the responsiveness and resistance to antiangiogenic therapy, in part by stimulating VEGF-independent pathways, as well as the production of angiogenic chemokines and cytokines^44^. In an ovarian cancer mouse model, the depletion of macrophages with CSF1R inhibitors restored sensitivity to bevacizumab and paclitaxel in the setting of adaptive resistance^45^. The addition of CSF1R inhibitors to standard chemotherapy or anti-angiogenic therapy may be an attractive option in the treatment of angiosarcoma that should be further investigated.

Our current study is limited by its retrospective single-institution design. Nonetheless, this study represents a large cohort of patients with data curated from a prospectively maintained database. The findings on systemic and local immune responses in angiosarcoma as well as their clinical correlates, while intriguing, remain hypothesis-generating and need to be validated in future studies.

In conclusion, the systemic and local immune responses may influence clinical outcomes and chemoresponsiveness in angiosarcomas. A deeper understanding of the immune and oncogenic signaling pathways involved in their pathobiology may provide clues to overcome chemoresistance and improve patient outcomes in this devastating disease.

## Methods

### Patient cohort

A total of 150 patients diagnosed with histologically proven angiosarcoma at the Singapore General Hospital (SGH) and National Cancer Centre Singapore (NCCS) between January 2000 and January 2020 were identified. All of the cases were reviewed by certified pathologists and the diagnoses were supported by immunohistochemical staining for vascular markers such as CD31 and/or ERG. Kaposi sarcoma, epithelioid hemangioendothelioma, and intimal sarcoma were excluded from the study. Median follow-up was 0.9 years for the whole cohort. Clinicopathological information available included sex, age, ethnicity, primary tumor site, performance status, cardiovascular risk factors, predisposing risk factors, presence of epithelioid histomorphology, presence of distant metastasis, tumor size, and type of treatment received. Age, sex, and ethnicity of the patients were corroborated against their National Registry Identification Cards. Tumor size was defined as either the largest diameter measured in resected pathological specimens or on CT imaging. Positive (R1) or negative (R0) surgical margins were defined depending upon microscopic involvement on histopathological analysis. These data were obtained at the time of diagnosis and at subsequent follow-up. Written informed consent for use of biospecimens and clinical data were obtained in accordance with the Declaration of Helsinki. This work was done under approval from the SingHealth Centralized Institution Review Board (CIRB 2018/3182). All methods were performed in accordance with the relevant guidelines and regulations. Clinicopathological characteristics of all patients with angiosarcoma are summarized in Table 1.

### Analysis of peripheral blood neutrophil-to-lymphocyte ratios (NLR)

A total of 112 patients with existing peripheral blood neutrophil and lymphocyte counts at the time of diagnosis (prior to any therapy including surgery) and/or metastatic relapse were analyzed. None of these patients had evidence of a hematological disorder or an infectious process at the time of blood sampling. The NLR was calculated by dividing absolute neutrophil counts by absolute lymphocyte counts from the same blood sample. The optimal cut-off for NLR (> 2.5) as a univariable predictor of OS was previously determined by Receiver operating characteristic (ROC) curve analysis and used in this study^16^.

### NanoString gene expression profiling

We used the NanoString PanCancer IO360 panel (NanoString Technologies, Seattle, WA, USA) to interrogate gene expression on FFPE tissue, following manufacturer’s protocol using the nCounter platform. Briefly, RNA was extracted from five 10 μm sections on all samples with adequate tumor tissue available and analyzed using the 2100 Bioanalyzer (Agilent Technologies, Palo Alto, CA, USA). After excluding samples with suboptimal RNA integrity and content, the remaining samples were included in the nCounter analysis. The final set of data available (*n* = 35) was analyzed on the nSolver 4.0 Advanced Analysis module using default settings to derive differentially expressed genes, pathway scores, and cell-type scores. A tumor neutrophil-to-lymphocyte ratio (tNLR) was obtained by dividing neutrophil scores by B- and T-lymphocyte scores combined. High tNLR was defined as more than 0.91 by ROC curve analysis against survival status as the dichotomous variable (AUC 0.626, 95% CI 0.447–0.783).

### Multiplex Immunohistochemistry/Immunofluorescence (mIHC/IF)

mIHC/IF was performed (*n* = 34) using an Opal Multiplex fIHC kit (Akoya Bioscience, Menlo Park, CA, USA), as previously described^5^. All cases profiled were also correspondingly profiled on the NanoString PanCancer IO360 panel and included in a combined analysis. Briefly, slides were labeled with PD-L1, CD68, CD8, Foxp3, CD15, and ERG, followed by appropriate secondary antibodies (detailed protocol previously reported^46^). Ten images of viable tumor regions that were selected randomly by pathologists were acquired for each case using a Vectra 3 pathology imaging system microscope (Akoya Bioscience, Menlo Park, CA, USA) then analyzed and scored by a pathologist. The number of immune cells scored were normalized against ERG+ (tumor) cells and log2 transformed prior to correlating with NanoString data.

### Statistics

Comparisons of the frequencies of categorical variables were performed using Pearson’s Chi-squared tests or Fisher’s exact test, as appropriate. Box-Whisker plots were used to represent continuous variables and Mann-Whitney *U* tests were used to evaluate potential associations with NLR levels. Pair-wise comparisons were made using the Wilcoxon signed-rank test. The primary and secondary survival endpoints are OS and relapse-free survival (RFS), respectively. RFS was defined as the time elapsed from the date of diagnosis till the date of relapse or death from any cause. OS was measured from the date of diagnosis till the date of death from any cause, or was censored at the date of the last follow-up for survivors. Locoregional failure was calculated as the time from diagnosis to the date of local or regional recurrence. Distant failure was calculated as the time from diagnosis to the date of distant recurrence. TTP was calculated from the start date of chemotherapy to first documented date of disease progression. Actuarial survival was estimated using Kaplan-Meier survival curves, and compared using log-rank tests. Hazard ratios (HR) with corresponding 95% confidence intervals (95% CI) of mortality were calculated using Cox proportional hazards regression. Multivariate Cox regression model via a backward procedure was used to test for independent factors identified on univariate analysis.

A prognostic scoring model incorporating the presence of metastasis at diagnosis, age >65 years, and high peripheral blood NLR (“MAN” score) was created. Each independently significant variable attributed a point, and a Kaplan-Meier survival curve was used to compare survival between patients scoring 0–1, 2, and 3 on the index. All statistical calculations were performed assuming a two-sided test with significance level of 0.05 unless otherwise stated. All tests were performed using MedCalc for Windows version 19.0.4 (MedCalc Software, Ostend, Belgium).

### Reporting summary

Further information on research design is available in the Nature Research Reporting Summary linked to this article.

## Supplementary information

Reporting summary.

Supplemental Appendix.

## Data Availability

The NanoString gene expression profiling data generated during the current study, are available in Gene Expression Omnibus^47^: https://identifiers.org/geo:GSE162370. All other datasets generated and analyzed during the study (including pathologist scores of the multiplex immunohistochemistry/immunofluorescence images, survival data, patient clinical data, and analysis of peripheral blood neutrophil-to-lymphocyte ratios), are not publicly available to protect patient privacy, but will be made available from the corresponding author on reasonable request. For data access requests, please contact Dr. Jason Yongsheng Chan, Division of Medical Oncology, National Cancer Centre Singapore, e-mail address: jason.chan.y.s@nccs.com.sg. The data generated and analyzed during this study are described in the following metadata record^48^: 10.6084/m9.figshare.13469544.
