# Supplementary material for: Clinical implications of systemic and local immune responses in human angiosarcoma
Source: NPJ Precis Oncol. 2021 Feb 12;5:11. doi: 10.1038/s41698-021-00150-x (PMC7881182; doi:10.1038/s41698-021-00150-x)
Supplement: Supplementary file 2 — Supplemental Appendix. [file 41698_2021_150_MOESM2_ESM.pdf]

SUPPLEMENTARY APPENDIX

Supplement to: Clinical implications of systemic and local immune responses in human angiosarcoma

CONTENTS

1.0 Supplementary Figures

Supplementary Figure 1. Survival outcomes of patients with localized angiosarcoma with or without curative surgery.
Supplementary Figure 2. Survival outcomes of patients with localized angiosarcoma undergoing curative therapy.
Supplementary Figure 3. Survival outcomes of patients with metastatic angiosarcoma.
Supplementary Figure 4. Correlation of NLR and absolute neutrophil/lymphocyte counts.
Supplementary Figure 5. Correlation of intratumoral NLR (tNLR) with tumor-infiltrating immune cells.
Supplementary Figure 6. Gene expression and chemotherapy response.

2.0 Supplementary Tables

Supplementary Table 1. Clinical features of patients with curatively resected non-metastatic angiosarcoma.
Supplementary Table 2. Univariate survival analysis for curatively resected non-metastatic angiosarcoma.
Supplementary Table 3. Multivariate survival analysis for curatively resected non-metastatic angiosarcoma.
Supplementary Table 4. Clinical features of patients with metastatic angiosarcoma.
Supplementary Table 5. Univariate survival analysis of patients with metastatic angiosarcoma.
Supplementary Table 6. Multivariate survival analysis of patients with metastatic angiosarcoma.
Supplementary Table 7. Clinical features of angiosarcoma and neutrophil-lymphocyte ratio (NLR) at diagnosis.
Supplementary Table 8. Univariate survival analysis of the study cohort with available NLR levels.
Supplementary Table 9. Multivariate survival analysis of the study cohort with available NLR levels.
Supplementary Table 10. Correlation of intratumoral NLR (tNLR) and cell-type scores with NanoString pathway scores.
Supplementary Table 11. Differentially expressed genes between non-responders and responders to chemotherapy.
Supplementary Table 12. Correlation of NanoString pathway scores with chemotherapy response.

1.0 Supplementary Figures

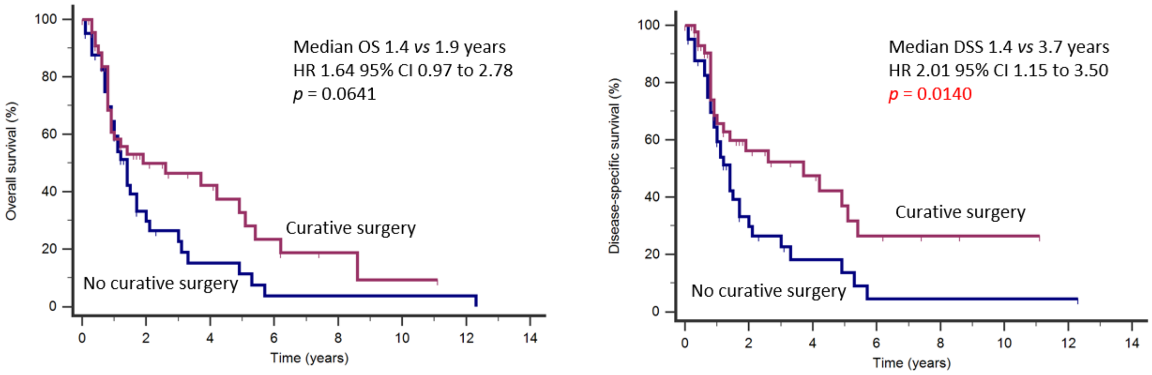

Supplementary Fig. 1 Survival outcomes of patients with localized angiosarcoma with or without curative surgery. Patients with localized angiosarcoma who did not undergo curative surgery did poorer in terms of OS and DSS.

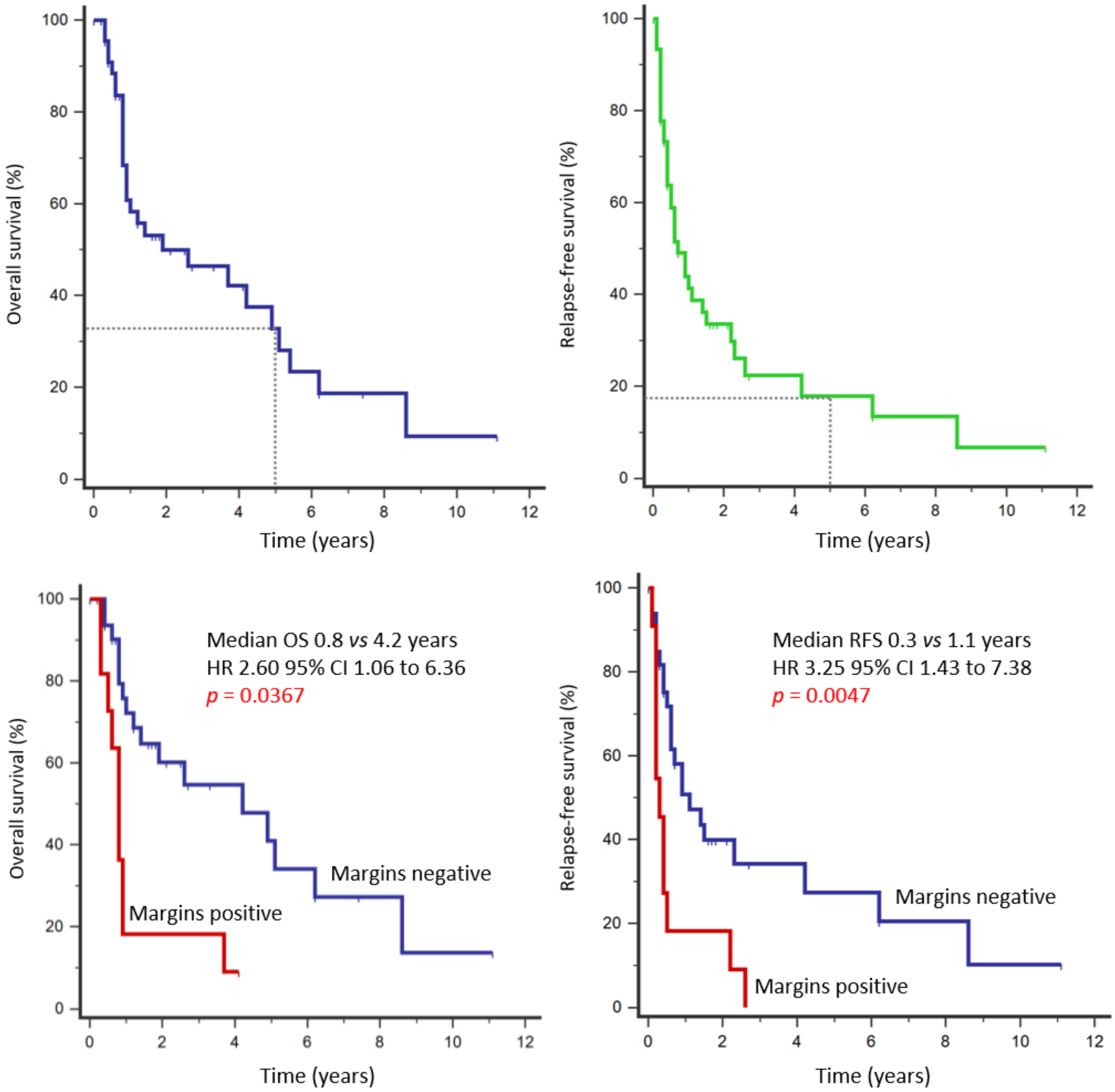

Supplementary Fig. 2 Survival outcomes of patients with localized angiosarcoma undergoing curative therapy. Median OS was 1.9 years and median RFS was 0.7 years. At 5 years, 32.8% of the patients remained alive, while 17.9% were relapse-free. The presence of microscopic positive margins was independently correlated with worse OS and RFS.

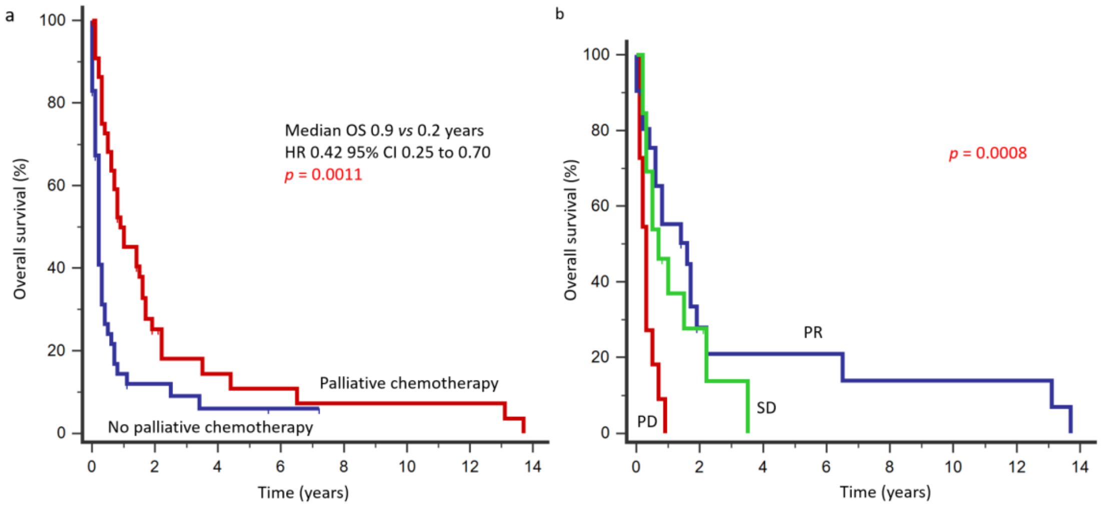

Supplementary Fig. 3 Survival outcomes of patients with metastatic angiosarcoma. a Patients who received palliative chemotherapy demonstrated improved OS compared to those who did not receive it. **b** Best responses obtained following palliative chemotherapy were significantly associated with OS.

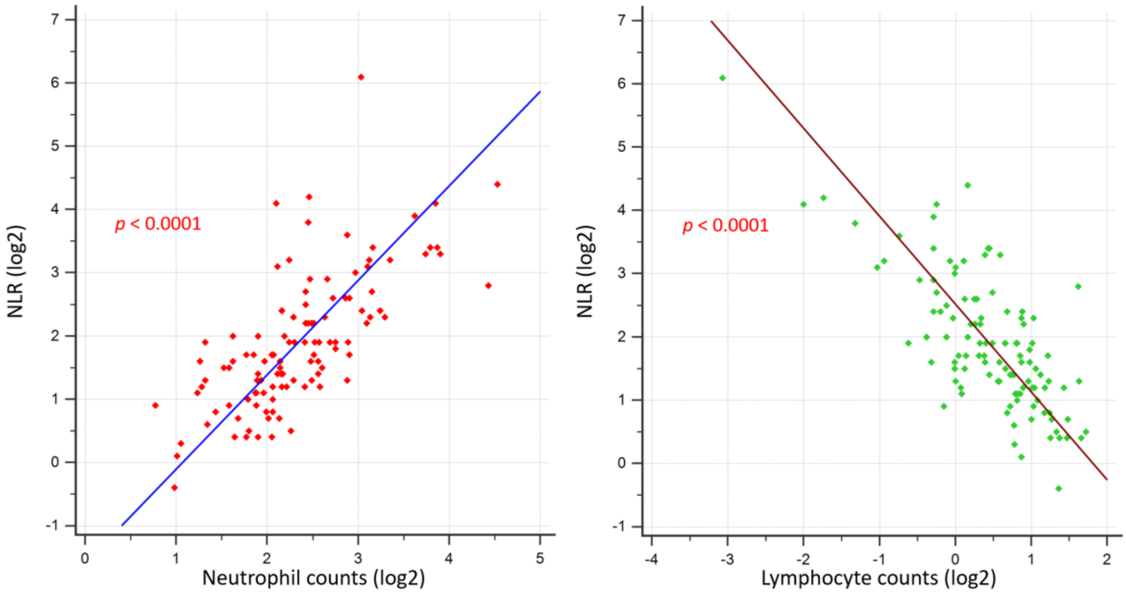

Supplementary Fig. 4 Correlation of NLR and absolute neutrophil/lymphocyte counts. Positive correlation was demonstrated between NLR and absolute neutrophil counts (Spearman's rho 0.720, 95% CI 0.617 to 0.799, $p < 0.0001$), while a converse correlation with absolute lymphocyte counts was found (Spearman's rho −0.683, 95% CI −0.771 to −0.570, $p < 0.0001$).

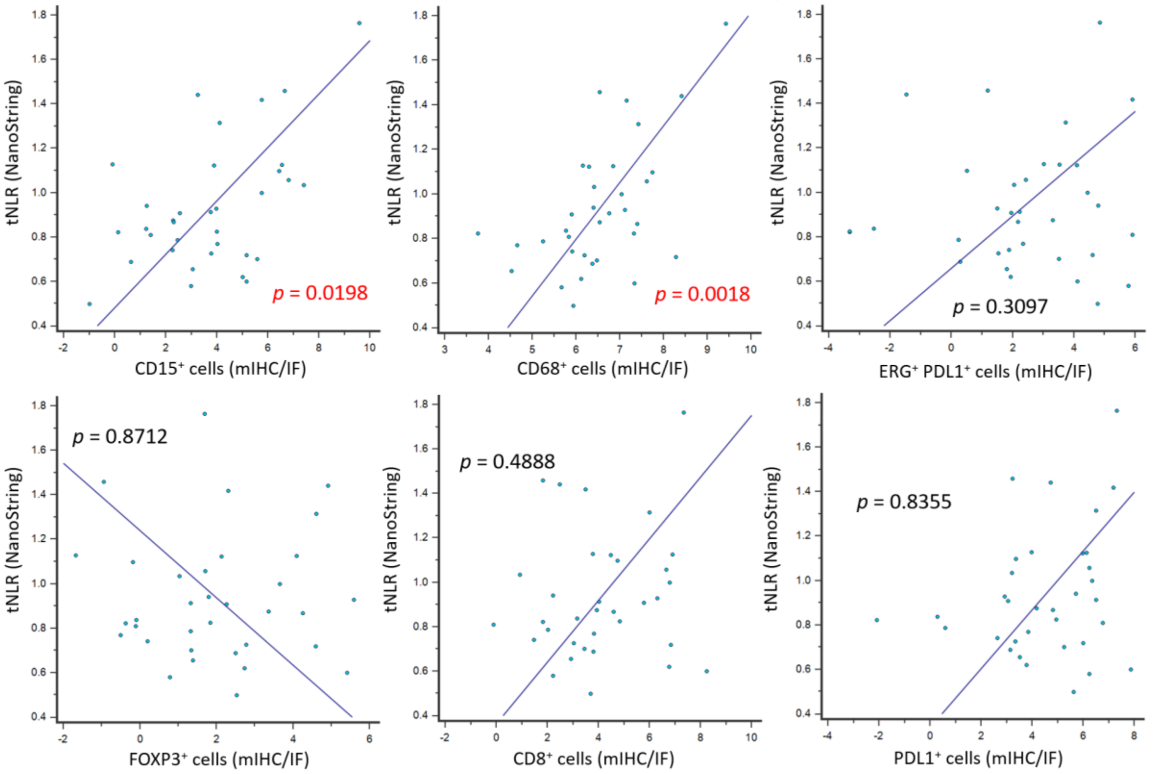

Supplementary Fig. 5 Correlation of intratumoral NLR (tNLR) with tumor-infiltrating immune cells. Tumor-infiltrating immune cells were visualized via Multiplex Immunohistochemistry/Immunofluorescence (mIHC/IF). The proportion of neutrophils (CD15+), macrophages (CD68+), cytotoxic T-cells (CD8+) and regulatory T-cells (FOXP3+) relative to tumor cells (ERG+) were obtained and correlated with log2-transformed ratios of tumor neutrophil to lymphocyte (tNLR) scores inferred from NanoString transcriptomic profiling.

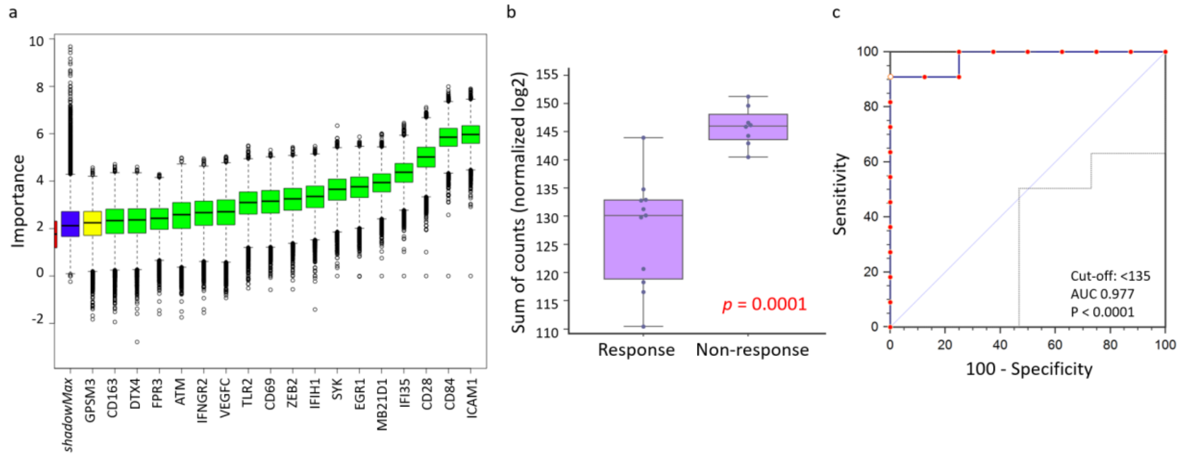

Supplementary Fig. 6 Gene expression and chemotherapy response. a A random-forest classification-based Boruta algorithm identified 17 important genes (all upregulated in non-responders). **b-c** Summation of their normalized log2 count data was able to predict chemotherapy response with a sensitivity of 90.9% and specificity of 100% at a cut-off of < 135.

Supplementary Table 1. Clinical features of patients with curatively resected non-metastatic angiosarcoma

Characteristic (n)	Tumor site (%)		*p*
	**AS-HN**	**Other**	
Total (48)	24 (50.0%)	24 (50.0%)	-
Sex			
Male (27)	21 (78.6%)	6 (21.4%)	**< 0.0001**
Female (21)	3 (14.3%)	18 (85.7%)	
Age at diagnosis (years)			
> 65 (20)	17 (85.7%)	3 (14.3%)	**< 0.0001**
≤ 65 (28)	7 (25.0%)	21 (75.0%)	
Ethnicity			
Chinese (39)	21 (55.0%)	18 (45.0%)	0.461
Other (9)	3 (33.3%)	6 (66.7%)	
Performance status (ECOG score)			
0 (36)	18 (50.0%)	18 (50.0%)	1.000
1-4 (9)	4 (44.4%)	5 (55.6%)	
Cardiovascular co-morbidities †			
Present (23)	17 (75.0%)	6 (25.0%)	**0.002**
Absent (25)	7 (28.0%)	18 (72.0%)	
Tumor size			
≥ 5 cm (20)	7 (35.0%)	13 (65.0%)	0.083
< 5 cm (19)	12 (63.2%)	7 (36.8%)	
Epithelioid component			
Present (26)	15 (57.7%)	11 (42.3%)	0.295
Absent (17)	7 (41.2%)	10 (58.8%)	
Surgical margins			
Positive (11)	6 (54.5%)	5 (45.5%)	0.734
Negative (35)	16 (45.7%)	19 (54.3%)	
Adjuvant radiation therapy			
Yes (22)	12 (54.5%)	10 (45.5%)	0.566
No (26)	12 (48.1%)	14 (51.9%)	

†Includes hypertension, hyperlipidemia, diabetes mellitus, ischemic heart disease, cerebrovascular disease

Data unavailable for ECOG score (n = 3), surgical margins (n = 2), tumor size (n = 9), histomorphology (n = 5)

Abbreviations: AS-HN, angiosarcoma of the head and neck

Supplementary Table 2. Univariate survival analysis for curatively resected non-metastatic angiosarcoma

	Overall survival		Disease-specific survival		Relapse-free survival		Locoregional failure		Distant failure	
**Characteristic**	**HR (95% CI)**	***p***	**HR (95% CI)**	***p***	**HR (95% CI)**	***p***	**HR (95% CI)**	***p***	**HR (95% CI)**	***p***
Sex (male *vs* female)	1.28 (0.59 to 2.78)	0.530	0.81 (0.33 to 1.94)	0.629	1.58 (0.79 to 3.18)	0.197	1.57 (0.62 to 4.00)	0.345	1.03 (0.44 to 2.44)	0.941
Age at diagnosis (> 65 *vs* ≤ 65 years)	1.50 (0.70 to 3.25)	0.299	1.25 (0.53 to 2.98)	0.613	1.76 (0.86 to 3.62)	0.123	**2.89 (1.10 to 7.58)**	**0.031**	1.26 (0.54 to 2.95)	0.592
Ethnicity (Chinese *vs* other)	1.76 (0.71 to 4.34)	0.221	1.30 (0.46 to 3.64)	0.622	1.52 (0.64 to 3.62)	0.348	1.08 (0.32 to 3.63)	0.903	1.30 (0.48 to 3.56)	0.607
Cardiovascular co-morbidities (Present *vs* absent)	0.91 (0.41 to 1.98)	0.803	0.75 (0.31 to 1.83)	0.531	1.20 (0.59 to 2.40)	0.616	1.81 (0.71 to 4.57)	0.213	1.09 (0.47 to 2.57)	0.839
Performance status (ECOG 1-4 *vs* 0)	2.73 (0.96 to 7.80)	0.061	**3.40 (1.05 to 11.00)**	**0.041**	2.13 (0.79 to 5.71)	0.135	1.59 (0.45 to 5.62)	0.475	2.25 (0.72 to 7.04)	0.164
Primary tumor site (AS-HN *vs* other)	0.84 (0.39 to 1.82)	0.659	0.53 (0.22 to 1.26)	0.151	1.12 (0.55 to 2.25)	0.759	2.07 (0.82 to 5.25)	0.124	0.64 (0.27 to 1.50)	0.304
Tumor size (≥ 5 cm *vs* < 5 cm)	**3.04 (1.25 to 7.38)**	**0.014**	**3.20 (1.17 to 8.73)**	**0.023**	**2.35 (1.02 to 5.38)**	**0.044**	1.08 (0.39 to 2.95)	0.882	**2.89 (1.04 to 8.02)**	**0.041**
Epithelioid component (Present *vs* absent)	1.21 0.52 to 2.81	0.661	1.06 0.41 to 2.71	0.907	1.67 0.79 to 3.50	0.178	1.88 0.74 to 4.80	0.186	1.16 0.47 to 2.86	0.753
Surgical margins (Positive *vs* negative)	**6.53 (2.15 to 19.86)**	**0.001**	**4.48 (1.29 to 15.61)**	**0.019**	**4.48 (1.65 to 12.14)**	**0.003**	**7.37 (2.12 to 25.60)**	**0.002**	1.49 (0.48 to 4.63)	0.487
Adjuvant radiation therapy (Yes *vs* no)	0.58 (0.27 to 1.25)	0.162	0.63 (0.26 to 1.50)	0.300	0.81 (0.40 to 1.62)	0.549	0.85 (0.33 to 2.15)	0.724	1.01 (0.43 to 2.35)	0.985

Abbreviations: AS-HN, angiosarcoma of the head and neck

Supplementary Table 3. Multivariate survival analysis for curatively resected non-metastatic angiosarcoma

	Overall survival		Relapse-free survival	
**Characteristic**	**HR (95% CI)**	***p***	**HR (95% CI)**	***p***
Surgical margins (Positive *vs* negative)	**2.60 (1.06 to 6.36)**	**0.037**	**3.25 (1.43 to 7.38)**	**0.005**

Supplementary Table 4. Clinical features of patients with metastatic angiosarcoma

Characteristic (n)	Tumor site (%)		*p*
	AS-HN	Other	
Total (94)	48 (51.1%)	46 (48.9%)	
Time of distant metastasis			
At relapse (34)	19 (55.9%)	15 (44.1%)	0.484
At diagnosis (60)	29 (48.3%)	31 (51.7%)	
Sex			
Male (61)	33 (54.1%)	28 (45.9%)	0.426
Female (33)	15 (45.5%)	18 (54.5%)	
Age at diagnosis of metastasis (years)			
> 65 (52)	38 (73.1%)	14 (26.9%)	**< 0.0001**
≤ 65 (42)	10 (23.8%)	32 (76.2%)	
Ethnicity			
Chinese (75)	40 (53.3%)	35 (46.7%)	0.384
Other (19)	8 (42.1%)	11 (57.9%)	
Performance status (ECOG score)			
0 (40)	17 (42.5%)	23 (57.5%)	0.243
1-4 (51)	28 (54.9%)	23 (45.1%)	
Cardiovascular co-morbidities †			
Present (57)	37 (64.9%)	20 (35.1%)	**0.001**
Absent (37)	11 (29.7%)	26 (70.3%)	
Epithelioid component			
Present (42)	15 (35.7%)	27 (64.3%)	0.105
Absent (33)	18 (54.5%)	15 (45.5%)	
Liver metastases			
Present (39)	20 (54.1%)	17 (45.9%)	0.642
Absent (55)	28 (49.1%)	29 (50.9%)	
Lung metastases			
Present (46)	29 (63.0%)	17 (33%)	**0.024**
Absent (48)	19 (39.6%)	29 (60.4%)	
Bone metastases			
Present (22)	10 (50.0%)	10 (50.0%)	0.915
Absent (72)	38 (51.4%)	36 (48.6%)	
Lymph node metastases			
Present (29)	24 (68.6%)	11 (31.4%)	**0.009**
Absent (65)	24 (40.7%)	35 (59.3%)	
Peritoneal/pleural metastases			
Present (21)	4 (19.0%)	17 (81.0%)	**0.001**
Absent (73)	44 (60.3%)	29 (39.7%)	
Splenic metastases			
Present (13)	7 (58.3%)	5 (41.7%)	0.759
Absent (81)	41 (50.0%)	41 (50.0%)	

†Includes hypertension, hyperlipidemia, diabetes mellitus, ischemic heart disease, cerebrovascular disease

Data unavailable for ECOG score (n = 3), histomorphology (n = 19)

Abbreviations: AS-HN, angiosarcoma of the head and neck

Supplementary Table 5. Univariate survival analysis of patients with metastatic angiosarcoma

	Overall survival	
**Characteristic**	**HR (95% CI)**	***p***
Sex<br>(male *vs* female)	0.98<br>(0.60 to 1.60)	0.939
Age at diagnosis of metastasis (years)<br>(> 65 *vs* ≤ 65 years)	**2.19**<br>**(1.36 to 3.52)**	**0.001**
Ethnicity<br>(Chinese *vs* other)	0.76<br>(0.39 to 1.47)	0.418
Cardiovascular co-morbidities<br>(Present *vs* absent)	1.30<br>(0.81 to 2.10)	0.276
Performance status<br>(ECOG 1-4 *vs* 0)	1.56<br>(0.96 to 2.53)	0.072
Tumor site<br>(AS-HN *vs* other)	1.09<br>(0.68 to 1.74)	0.713
Epithelioid component<br>(Present *vs* absent)	1.25<br>(0.73 to 2.14)	0.411
Time of distant metastasis<br>(At relapse *vs* at diagnosis)	**1.81**<br>**(1.06 to 3.07)**	**0.029**
Liver metastases<br>(Present *vs* absent)	**1.92**<br>**(1.15 to 3.22)**	**0.013**
Lung metastases<br>(Present *vs* absent)	1.57<br>(0.97 to 2.55)	0.067
Bone metastases<br>(Present *vs* absent)	**0.49**<br>**(0.29 to 0.83)**	**0.007**
Lymph node metastases<br>(Present *vs* absent)	1.05<br>(0.65 to 1.71)	0.841
Peritoneal/pleural metastases<br>(Present *vs* absent)	**2.45**<br>**(1.27 to 4.75)**	**0.008**
Palliative chemotherapy<br>(Yes *vs* no)	**0.40**<br>**(0.24 to 0.67)**	**0.000**

Abbreviations: AS-HN, angiosarcoma of the head and neck

Supplementary Table 6. Multivariate survival analysis of patients with metastatic angiosarcoma

	Overall survival	
**Characteristic**	**HR (95% CI)**	***p***
Age at diagnosis of metastasis (years)<br>(> 65 *vs* ≤ 65 years)	**1.92**<br>**(1.15 to 3.23)**	**0.013**
Performance status<br>(ECOG 1-4 *vs* 0)	**1.74**<br>**(1.01 to 3.02)**	**0.048**
Time of distant metastasis<br>(At relapse *vs* at diagnosis)	**1.82**<br>**(1.02 to 3.24)**	**0.041**
Bone metastases<br>(Present *vs* absent)	**0.50**<br>**(0.26 to 0.97)**	**0.039**
Liver metastases<br>(Present *vs* absent)	**1.94**<br>**(1.17 to 3.21)**	**0.010**
Peritoneal/pleural metastases<br>(Present *vs* absent)	**1.74**<br>**(1.00 to 3.04)**	**0.050**
Palliative chemotherapy<br>(Yes *vs* no)	**0.42**<br>**(0.25 to 0.70)**	**0.001**

Supplementary Table 7. Clinical features of angiosarcoma and neutrophil-lymphocyte ratio (NLR) at diagnosis

Characteristic (N)	NLR > 2.5	NLR ≤ 2.5	*p*
Total (112)	78 (69.6%)	34 (30.4%)	-
Sex			
Male (70)	52 (74.3%)	18 (25.7%)	0.170
Female (42)	26 (61.9%)	16 (38.1%)	
Age at diagnosis (years)			
> 65 (62)	46 (74.2%)	16 (25.8%)	0.246
≤ 65 (50)	32 (64.0%)	18 (36.0%)	
Ethnicity			
Chinese (94)	66 (70.2%)	28 (29.8%)	0.765
Other (18)	12 (66.7%)	6 (33.3%)	
Performance status (ECOG score)			
0 (57)	35 (61.4%)	22 (38.6%)	0.055
1-4 (55)	43 (78.2%)	12 (21.8%)	
Cardiovascular co-morbidities [†]			
Present (69)	50 (72.5%)	19 (27.5%)	0.413
Absent (43)	28 (65.1%)	15 (34.9%)	
Etiology			
Primary (95)	68 (71.6%)	27 (28.4%)	0.294
Secondary (17)	10 (58.8%)	7 (41.2%)	
Epithelioid component			
Present (50)	36 (72.0%)	14 (28.0%)	0.741
Absent (45)	31 (68.9%)	14 (31.1%)	
Distant metastasis at diagnosis			
Present (48)	37 (77.1%)	11 (22.9%)	0.140
Absent (64)	41 (64.1%)	23 (35.9%)	
Primary tumor site			
AS-HN (64)	46 (71.9%)	18 (28.1%)	0.555
Other (48)	32 (66.7%)	16 (33.3%)	

[†]Includes hypertension, hyperlipidemia, diabetes mellitus, ischemic heart disease, cerebrovascular disease

Abbreviations: AS-HN, angiosarcoma of the head and neck

Data unavailable for histomorphology (n = 17)

Supplementary Table 8. Univariate survival analysis of the study cohort with available NLR levels

	Overall survival	
**Characteristic**	**HR (95% CI)**	***p***
NLR<br>(> 2.5 *vs* ≤ 2.5)	**1.84**<br>**(1.18 to 2.87)**	**0.007**
Sex<br>(male *vs* female)	1.14<br>(0.74 to 1.76)	0.549
Age at diagnosis<br>(> 65 *vs* ≤ 65 years)	**1.77**<br>**(1.15 to 2.71)**	**0.009**
Ethnicity<br>(Chinese *vs* other)	1.15<br>(0.64 to 2.09)	0.638
Cardiovascular co-morbidities<br>(Present *vs* absent)	1.30<br>(0.85 to 2.00)	0.230
Performance status<br>(ECOG 1-4 *vs* 0)	**1.87**<br>**(1.21 to 2.88)**	**0.005**
Primary tumor site<br>(AS-HN *vs* other)	0.86<br>(0.55 to 1.33)	0.494
Etiology<br>(Secondary *vs* primary)	1.00<br>(0.56 to 1.81)	0.992
Epithelioid component<br>(Present *vs* absent)	1.53<br>(0.96 to 2.46)	0.076
Distant metastasis at diagnosis<br>(Present *vs* absent)	**1.88**<br>**(1.19 to 2.97)**	**0.007**

Abbreviations: AS-HN, angiosarcoma of the head and neck

Supplementary Table 9. Multivariate survival analysis of the study cohort with available NLR levels

	Overall survival	
**Characteristic**	**HR (95% CI)**	***p***
NLR<br>(> 2.5 *vs* ≤ 2.5)	**1.79**<br>**(1.09 to 2.92)**	**0.021**
Age at diagnosis<br>(> 65 *vs* ≤ 65 years)	**1.80**<br>**(1.16 to 2.79)**	**0.008**
Distant metastasis at diagnosis<br>(Present *vs* absent)	**1.81**<br>**(1.18 to 2.77)**	**0.006**

Supplementary Table 10. Correlation of intratumoral NLR (tNLR) and cell-type scores with NanoString pathway scores

NanoString-derived cell types	Neutrophil-Lymphocyte ratio (tNLR)		Neutrophil score		Lymphocyte score		Macrophage score	
Pathway/Cell-type	Spearman's rho (95% CI)	p-value	Spearman's rho (95% CI)	p-value	Spearman's rho (95% CI)	p-value	Spearman's rho (95% CI)	p-value
**Angiogenesis**	**0.664 0.425 to 0.817**	**<0.0001**	**0.587 0.316 to 0.770**	**0.0002**	-0.252 -0.540 to 0.0892	0.145	**0.617 0.358 to 0.788**	**0.0001**
**Matrix Remodeling and Metastasis**	**0.666 0.427 to 0.817**	**<0.0001**	**0.620 0.362 to 0.790**	**0.0001**	-0.168 -0.475 to 0.175	0.334	**0.721 0.511 to 0.850**	**<0.0001**
**Cytokine and Chemokine Signaling**	**0.538 0.250 to 0.739**	**0.0009**	**0.848 0.717 to 0.921**	**<0.0001**	0.302 -0.0345 to 0.577	0.078	**0.761 0.574 to 0.873**	**<0.0001**
**Myeloid Compartment**	**0.689 0.462 to 0.832**	**<0.0001**	**0.955 0.913 to 0.977**	**<0.0001**	0.213 -0.129 to 0.510	0.219	**0.770 0.587 to 0.878**	**<0.0001**
**Hypoxia**	**0.543 0.256 to 0.742**	**0.001**	0.413 0.0927 to 0.656	0.014	-0.339 -0.604 to -0.00704	0.046	**0.545 0.259 to 0.743**	**0.0007**
**Antigen Presentation**	0.150 -0.193 to 0.460	0.389	0.424 0.105 to 0.663	0.011	0.374 0.0462 to 0.629	0.027	**0.771 0.590 to 0.879**	**<0.0001**
**Cytotoxicity**	0.164 -0.179 to 0.471	0.347	0.462 0.152 to 0.689	0.005	0.387 0.0615 to 0.638	0.022	**0.767 0.583 to 0.876**	**<0.0001**
**Interferon Signaling**	0.300 -0.0373 to 0.575	0.080	0.475 0.169 to 0.698	0.004	0.181 -0.162 to 0.485	0.298	**0.819 0.669 to 0.905**	**<0.0001**
**Costimulatory Signaling**	0.058 -0.281 to 0.384	0.742	0.439 0.124 to 0.674	0.008	**0.525 0.233 to 0.731**	**0.001**	**0.684 0.454 to 0.828**	**<0.0001**
**Lymphoid Compartment**	-0.288 -0.567 to 0.0504	0.094	0.308 -0.0277 to 0.582	0.072	**0.901 0.811 to 0.949**	**<0.0001**	0.380 0.0540 to 0.633	0.024
**NF-kappaB Signaling**	-0.217 -0.513 to 0.126	0.212	0.262 -0.0785 to 0.547	0.129	**0.681 0.450 to 0.827**	**<0.0001**	0.0563 -0.282 to 0.382	0.748
**Immune Cell Adhesion and Migration**	0.222 -0.121 to 0.517	0.201	**0.522 0.229 to 0.729**	**0.001**	0.400 0.0767 to 0.647	0.017	**0.825 0.679 to 0.909**	**<0.0001**
**JAK-STAT Signaling**	0.387 0.0612 to 0.638	0.022	**0.764 0.577 to 0.874**	**<0.0001**	0.408 0.0863 to 0.652	0.015	**0.794 0.627 to 0.891**	**<0.0001**
**Apoptosis**	0.183 -0.160 to 0.486	0.294	0.049 -0.289 to 0.376	0.780	-0.227 -0.521 to 0.115	0.189	0.396 0.0720 to 0.644	0.019
**Metabolic Stress**	0.329 -0.00463 to 0.597	0.054	0.178 -0.165 to 0.482	0.307	-0.307 -0.581 to 0.0289	0.073	0.462 0.152 to 0.689	0.005
**Notch Signaling**	-0.210 -0.508 to 0.133	0.226	-0.156 -0.465 to 0.187	0.372	0.197 -0.146 to 0.498	0.257	-0.448 -0.680 to -0.135	0.007
**MAPK**	-0.145 -0.456 to 0.198	0.406	0.118 -0.224 to 0.434	0.499	0.352 0.0207 to 0.613	0.038	-0.180 -0.484 to 0.163	0.301
**PI3K-Akt**	-0.170 -0.476 to 0.173	0.330	0.087 -0.253 to 0.409	0.619	0.373 0.0449 to 0.628	0.028	-0.231 -0.524 to 0.110	0.181
**Wnt Signaling**	-0.248 -0.537 to 0.0924	0.150	0.019 -0.316 to 0.350	0.912	0.350 0.0194 to 0.612	0.039	-0.320 -0.590 to 0.0150	0.061
**Autophagy**	-0.151 -0.461 to 0.192	0.388	0.028 -0.309 to 0.357	0.876	0.281 -0.0580 to 0.561	0.102	-0.316 -0.587 to 0.0193	0.064
**Cell Proliferation**	0.098 -0.243 to 0.418	0.575	0.185 -0.158 to 0.488	0.288	0.124 -0.218 to 0.439	0.478	-0.228 -0.522 to 0.114	0.187
**DNA Damage Repair**	-0.0944 -0.415 to 0.247	0.590	0.102 -0.239 to 0.421	0.560	0.299 -0.0379 to 0.575	0.081	-0.292 -0.570 to 0.0461	0.089
**Epigenetic Regulation**	-0.063 -0.388 to 0.276	0.720	0.121 -0.221 to 0.436	0.490	0.263 -0.0770 to 0.548	0.127	-0.213 -0.510 to 0.130	0.220
**Hedgehog Signaling**	-0.190 -0.492 to 0.153	0.274	0.012 -0.322 to 0.344	0.944	0.268 -0.0722 to 0.552	0.120	-0.328 -0.596 to 0.00557	0.054
**TGF-beta Signaling**	0.248 -0.0933 to 0.537	0.152	0.070 -0.270 to 0.394	0.692	-0.315 -0.587 to 0.0206	0.066	0.319 -0.0159 to 0.590	0.062

Supplementary Table 11. Differentially expressed genes between non-responders and responders to chemotherapy			
Gene Symbol	log2 fold-change	LogfoldchangeStandard Error	Unadjusted p-value
FCGR2B	1.5	0.336	0.000
CD28	1.39	0.323	0.000
IFNGR2	0.932	0.237	0.001
SPP1	3.04	0.778	0.001
IFI35	1.13	0.297	0.001
RASAL1	-2.35	0.613	0.001
COL11A1	2.71	0.736	0.002
CD84	1.31	0.367	0.002
DTX4	-1.67	0.486	0.003
CD69	1.82	0.533	0.003
RSAD2	1.88	0.557	0.004
CCL8	1.48	0.44	0.004
CCL4	2.23	0.663	0.004
CCL5	1.85	0.554	0.004
SAMD9	1.49	0.445	0.004
IFIH1	1.26	0.381	0.004
CD79A	2.4	0.737	0.005
CXCR4	1.64	0.51	0.005
TRAF1	1.21	0.377	0.005
VEGFC	1.22	0.38	0.005
IFIT1	1.66	0.523	0.006
CD48	1.74	0.552	0.006
TWF1	0.814	0.258	0.006
CDH11	1.23	0.392	0.006
EGR1	1.82	0.583	0.006
SYK	0.774	0.25	0.007
ITGA4	1.19	0.393	0.008
CCL7	2.79	0.919	0.008
IL7R	1.63	0.545	0.008
ERO1A	1.48	0.494	0.008
FOSL1	1.77	0.594	0.008
KAT2B	0.542	0.183	0.009
CCL3/L1	2.1	0.719	0.009
MAP3K5	0.912	0.32	0.011
ZEB1	0.602	0.211	0.011
IRF5	0.984	0.35	0.012
ATM	0.953	0.341	0.013
CXCL8	5.29	1.9	0.013
C7	2.52	0.909	0.013
ITGA2	1.37	0.498	0.014
KLRB1	1.69	0.616	0.014
FPR3	1.04	0.381	0.014
ATF3	1.89	0.696	0.015
NRAS	0.839	0.309	0.015
IFI6	1.25	0.464	0.016
LAMB3	1.66	0.619	0.016
STAT2	0.5	0.186	0.016
ISG15	1.04	0.388	0.016
EIF2AK2	0.81	0.303	0.016
MB21D1	1.17	0.439	0.016
CLEC5A	1.77	0.669	0.017
IFIT3	1.33	0.506	0.018
NFKB1	0.376	0.143	0.018
IL6R	0.829	0.316	0.018
HLA-DMB	1.01	0.387	0.019
ZEB2	0.776	0.3	0.019
TREM2	1.8	0.698	0.019
FAP	1.54	0.594	0.019
OAS2	1.06	0.414	0.020
VEGFB	0.549	0.214	0.020
MS4A4A	1.1	0.43	0.020
OAS1	0.825	0.322	0.020
TGFBR1	0.909	0.355	0.020
VCAM1	1.21	0.474	0.020
MYD88	0.643	0.253	0.021
SGK1	0.798	0.315	0.022
ICAM1	1.23	0.486	0.022
CD45RA	1.52	0.604	0.022
CHUK	1.14	0.456	0.022

HERC6	1.14	0.453	0.022
MAML2	0.439	0.176	0.023
SLAMF7	1.22	0.492	0.024
TLR2	1.32	0.533	0.024
IFIT2	1.26	0.508	0.024
CD163	0.957	0.387	0.024
ITGAL	0.988	0.4	0.025
NKG7	1.29	0.525	0.025
IL22RA1	-1.55	0.63	0.025
CD27	1.35	0.55	0.025
ICAM3	0.623	0.255	0.026
DUSP2	1.45	0.596	0.026
MICB	0.808	0.333	0.027
TLR4	0.522	0.218	0.028
IL1B	1.93	0.81	0.029
TYMP	0.859	0.361	0.029
TBXAS1	1.2	0.506	0.030
MX1	1.34	0.567	0.030
CD300A	1.25	0.531	0.031
APOL6	0.861	0.368	0.032
CMKLR1	0.519	0.222	0.032
CASP1	0.774	0.333	0.033
CCL18	1.33	0.575	0.033
AKT1	0.558	0.241	0.034
GPSM3	0.672	0.293	0.035
APOE	1.14	0.5	0.036
PVRIG	1.5	0.66	0.036
C1QB	0.97	0.426	0.036
TREM1	3.02	1.33	0.036
CNTFR	-1.95	0.852	0.036
SREBF1	-0.673	0.298	0.037
PRKACB	1.14	0.51	0.039
TNFRSF1A	0.521	0.233	0.039
EXO1	-1.2	0.538	0.040
CDKN1A	0.653	0.294	0.040
HSD11B1	-1.65	0.747	0.042
TLR1	1.07	0.488	0.042
CD276	0.743	0.338	0.042
NEIL1	-1.2	0.551	0.044
CDC20	-0.876	0.404	0.045
NT5E	0.928	0.429	0.045
KRAS	0.74	0.343	0.045
NF1	0.462	0.214	0.046
IFNGR1	0.409	0.19	0.046
FLNB	0.697	0.324	0.046
FCGR2A	1.12	0.523	0.047
DTX3L	0.735	0.346	0.049
KLRK1	1.63	0.77	0.049
OLFML2B	0.993	0.469	0.049
CD247	1.49	0.704	0.050
PLA2G2A	-1.85	0.876	0.050
CD3E	0.924	0.438	0.050
IL1RN	1.49	0.709	0.050
E2F3	0.54	0.257	0.051
PPARGC1B	-1.19	0.569	0.051
TNFSF12	0.81	0.387	0.051
CASP3	0.77	0.368	0.052
HMGA1	1.07	0.513	0.053
LILRB4	0.826	0.397	0.053
IRF7	0.511	0.248	0.055
IRF1	0.626	0.304	0.056
PTPRC	1.18	0.574	0.056
PDCD1	-1.3	0.633	0.056
CD45RB	1.12	0.551	0.058
OASL	1.24	0.612	0.059
IRF3	0.656	0.324	0.059
ITGAM	0.949	0.469	0.059
C2	0.673	0.334	0.060
MMP7	-1.51	0.75	0.060
TIGIT	1.14	0.568	0.060
JAK2	0.554	0.275	0.060

CXorf36	-1.1	0.55	0.062
HLA-DMA	0.82	0.41	0.062
DDB2	1.1	0.551	0.062
LTBP1	1.3	0.653	0.063
CD19	1.34	0.672	0.064
CD6	1.28	0.646	0.064
BCAT1	1.12	0.567	0.064
PIK3CD	0.749	0.379	0.065
IL2RB	0.785	0.397	0.065
CTLA4	-1.62	0.819	0.065
CD38	1.15	0.585	0.066
GBP2	0.843	0.431	0.067
PIK3R5	0.739	0.379	0.068
GZMK	1.25	0.641	0.069
CYBB	0.723	0.372	0.069
CXCL13	1.58	0.813	0.069
GZMA	1.74	0.898	0.069
ZAP70	1.09	0.563	0.070
GLUL	0.635	0.329	0.070
LAMC2	1.72	0.895	0.072
RIPK3	0.49	0.256	0.072
IL33	-0.906	0.473	0.073
TNFRSF9	1.52	0.795	0.073
HAVCR2	0.937	0.493	0.074
MRE11	0.588	0.309	0.074
ADAM12	1.32	0.696	0.075
THBS1	1.11	0.59	0.077
PCK2	-0.717	0.381	0.077
COL11A2	-1.5	0.795	0.077
COMP	1.51	0.814	0.081
HK2	0.829	0.447	0.081
SAMSN1	1.25	0.676	0.081
CTSS	1.09	0.588	0.082
EIF4EBP1	0.775	0.421	0.083
STC1	1.38	0.756	0.085
RUNX3	0.715	0.395	0.088
SRP54	0.458	0.254	0.089
PIK3CA	0.513	0.284	0.089
CD45RO	1.16	0.647	0.090
FCGR1A	1.27	0.712	0.091
CD14	0.622	0.351	0.095
CD96	1.02	0.579	0.095
PTEN	0.532	0.302	0.097
LILRA1	-1.25	0.708	0.097
HLA-DPA1	0.955	0.546	0.099
BCL2L1	0.388	0.223	0.100
DUSP1	1.08	0.627	0.103
IL6	2.12	1.24	0.105
P4HA1	0.822	0.483	0.107
CLEC14A	0.704	0.414	0.107
SIGLEC1	0.851	0.503	0.109
SH2D1A	0.906	0.536	0.109
HLA-B	0.713	0.424	0.111
UBE2T	-0.782	0.465	0.111
GBP1	1.2	0.712	0.111
CD3D	1.2	0.718	0.112
PDK1	0.802	0.479	0.112
ITGB2	0.837	0.502	0.114
MARCO	0.975	0.586	0.115
CXCL1	1.39	0.841	0.116
EDN1	0.834	0.504	0.117
IFI27	0.915	0.555	0.118
ROCK1	0.525	0.319	0.118
CDK6	0.407	0.247	0.118
CD8A	0.903	0.55	0.119
TNFAIP3	0.75	0.457	0.119
EPM2AIP1	0.17	0.105	0.123
LY96	0.85	0.527	0.125
CD47	0.438	0.273	0.126
ITGAV	0.417	0.259	0.127
CDK2	0.513	0.321	0.128

NOTCH2	0.677	0.423	0.128
C1QA	0.695	0.436	0.129
LDHB	0.697	0.439	0.130
KLRD1	0.9	0.566	0.130
AREG	1.19	0.747	0.131
LDHA	0.903	0.572	0.133
CD68	0.747	0.475	0.134
PARP9	0.962	0.611	0.134
TDO2	1.45	0.923	0.134
MFGE8	0.654	0.417	0.136
KDR	-1.34	0.858	0.136
PDCD1LG2	1.06	0.679	0.138
IL32	0.902	0.581	0.139
MET	0.731	0.472	0.140
FASLG	1.24	0.8	0.140
IGF2R	0.189	0.123	0.143
JAK3	0.568	0.37	0.143
PKM	0.602	0.395	0.146
P4HA2	1.11	0.731	0.147
TNKS	0.425	0.28	0.148
PSMB10	0.397	0.262	0.148
SFRP4	2.12	1.4	0.148
CD86	0.815	0.539	0.149
IL34	-0.928	0.614	0.149
CASP8	0.522	0.346	0.149
PSMB8	0.254	0.169	0.151
GLI1	-1.3	0.867	0.152
IL16	0.539	0.36	0.152
FSTL3	0.898	0.6	0.152
PRKCA	0.662	0.445	0.155
CD2	0.833	0.56	0.155
APLNR	-1.23	0.825	0.156
RNLS	-0.651	0.44	0.158
NCAM1	1.03	0.699	0.158
IL1A	1.16	0.787	0.159
WNT2	1.18	0.804	0.160
BCL2	-0.582	0.397	0.161
CCL2	0.742	0.508	0.162
CXCL14	-1.26	0.865	0.162
IL24	1.38	0.947	0.162
IL10RA	0.382	0.263	0.164
RIPK2	0.767	0.53	0.166
MYC	-1.11	0.769	0.167
CD5	0.893	0.619	0.167
HLA-DOA	0.716	0.497	0.168
CCND2	0.579	0.403	0.169
FAM30A	1.15	0.804	0.170
ULBP2	-0.996	0.698	0.172
PSMB9	0.725	0.508	0.172
FCGRT	0.388	0.273	0.173
SERPINA1	1.06	0.749	0.176
SBNO2	0.461	0.327	0.177
ROR2	0.673	0.478	0.178
PTGER4	0.564	0.402	0.178
ITGA6	0.638	0.454	0.179
MELK	0.672	0.479	0.179
CD44	0.653	0.466	0.179
HLA-DPB1	0.837	0.598	0.180
FCGR3A/B	0.899	0.645	0.181
GNLY	0.825	0.594	0.183
RAD50	0.304	0.22	0.184
GLUD1	0.359	0.26	0.185
TYMS	-0.676	0.49	0.185
NFAM1	0.625	0.454	0.187
HLA-DRA	0.799	0.581	0.187
CDH2	0.952	0.695	0.189
RELB	0.337	0.247	0.189
FADD	0.348	0.255	0.189
IFI16	0.835	0.612	0.190
CD74	0.567	0.417	0.191
PLA1A	-1.68	1.24	0.193

DAB2	0.307	0.227	0.194
RICTOR	0.339	0.251	0.195
TRAT1	1.17	0.864	0.195
SPIB	0.811	0.6	0.195
CD4	0.339	0.253	0.198
IFITM1	0.506	0.38	0.200
MAGEB2	-1.07	0.804	0.202
STAT4	0.594	0.449	0.203
TLR3	0.824	0.623	0.204
TNFRSF11B	0.992	0.752	0.205
IRF9	0.542	0.411	0.205
NDUFA4L2	0.806	0.612	0.205
ITGB3	0.681	0.519	0.207
TWIST1	0.778	0.595	0.209
IFNAR1	0.715	0.55	0.211
B2M	0.694	0.535	0.212
PLOD2	1.03	0.792	0.213
UBA7	0.596	0.463	0.215
TNFRSF11A	-0.931	0.723	0.216
POLD1	0.552	0.431	0.217
TAPBPL	-0.271	0.212	0.220
XCL1/2	-0.996	0.783	0.222
CD70	-1.16	0.919	0.223
BAX	0.333	0.264	0.225
MRC1	0.748	0.595	0.226
RELA	0.444	0.354	0.227
CRABP2	0.821	0.66	0.231
ACVR1C	0.783	0.629	0.231
CCL22	-0.66	0.531	0.231
STAT1	0.514	0.416	0.234
CSF1	0.44	0.357	0.235
TNFRSF14	-0.452	0.368	0.236
IL11	1.47	1.2	0.238
ARG1	-1.77	1.46	0.240
COL5A1	0.724	0.595	0.240
FGF18	1.01	0.828	0.240
RB1	0.617	0.508	0.242
TMEM173	1.16	0.965	0.244
TPM1	0.587	0.487	0.244
IL2RA	0.519	0.433	0.247
LAG3	0.778	0.654	0.251
RBL2	0.303	0.255	0.252
CCR5	0.734	0.622	0.254
PARP4	0.308	0.262	0.255
CCND3	0.471	0.401	0.256
API5	0.181	0.155	0.258
TGFB1	0.666	0.574	0.262
H2AFX	-0.368	0.318	0.262
LIF	-0.99	0.855	0.263
DPP4	0.538	0.465	0.264
WNT2B	-0.607	0.527	0.266
TIE1	-0.661	0.577	0.267
PFKFB3	0.645	0.564	0.269
ADORA2A	0.54	0.472	0.269
PRF1	0.542	0.475	0.270
S100A12	0.954	0.838	0.271
S100A9	1.91	1.68	0.271
IL2RG	0.599	0.534	0.278
CLEC7A	0.674	0.603	0.279
ANLN	-0.465	0.419	0.282
FUT4	-0.542	0.488	0.282
TNFSF13	0.539	0.486	0.283
CXCR6	0.762	0.69	0.284
BIRC3	0.957	0.871	0.288
CXCL2	0.918	0.837	0.288
OAS3	0.37	0.337	0.289
ROBO4	0.676	0.619	0.290
AXL	0.544	0.499	0.291
BCL6B	-0.521	0.48	0.293
EIF2B4	-0.244	0.224	0.293
LAMA1	-0.889	0.818	0.293

TP53	-0.254	0.234	0.294
LCK	0.733	0.678	0.295
RELN	-1.4	1.29	0.296
CCR2	-0.544	0.509	0.300
CSF1R	0.634	0.596	0.302
PTCD2	-0.315	0.298	0.305
CDH1	-1.68	1.59	0.305
HMGB1	0.408	0.388	0.307
GBP4	0.782	0.743	0.307
HLA-F	0.585	0.556	0.308
DLL4	-0.67	0.638	0.308
LAIR1	0.436	0.416	0.310
CD7	1.06	1.01	0.312
CXCL16	0.559	0.536	0.312
CXCL10	1.56	1.5	0.314
ENO1	0.522	0.503	0.314
MS4A1	0.726	0.704	0.317
TAF3	-1.02	0.988	0.318
SLC16A1	0.458	0.446	0.319
HLA-DQA1	-2.4	2.36	0.324
RIPK1	0.177	0.176	0.328
LILRA3	-0.711	0.706	0.328
PDZK1IP1	0.906	0.902	0.329
MKI67	-0.388	0.387	0.330
WNT5A	0.618	0.617	0.331
DEPTOR	0.476	0.48	0.336
ZC3H12A	0.578	0.59	0.342
SIGLEC5	-0.921	0.943	0.343
CD1C	-0.726	0.749	0.346
NFKB2	0.371	0.387	0.351
HELLS	-0.42	0.439	0.352
TTC30A	-0.702	0.734	0.353
VHL	-0.44	0.461	0.353
FCAR	1.14	1.2	0.355
CDKN2A	-0.617	0.65	0.357
CCL20	1.17	1.24	0.358
P2RY13	0.383	0.407	0.359
IL21R	1.05	1.11	0.362
RPL7A	0.254	0.271	0.362
TRIM21	0.369	0.395	0.363
GPR160	0.411	0.441	0.364
MMRN2	-0.449	0.482	0.365
ID4	0.48	0.517	0.366
APH1B	0.58	0.628	0.369
ITGAX	0.594	0.644	0.369
TSLP	-0.841	0.911	0.370
MMP9	0.564	0.617	0.374
CX3CR1	-0.408	0.447	0.375
LGALS9	0.352	0.387	0.377
HDAC3	-0.176	0.195	0.380
ELOB	-0.311	0.346	0.381
AQP9	0.718	0.8	0.382
MAGEA12	-0.725	0.81	0.384
HLA-DQB1	-1.74	1.95	0.384
IKBKB	0.489	0.548	0.385
MTOR	-0.202	0.226	0.385
BBC3	0.325	0.365	0.386
NBN	0.421	0.475	0.387
PROM1	-0.839	0.946	0.388
NOTCH1	-0.355	0.401	0.388
IRF2	0.276	0.314	0.391
AXIN1	0.361	0.411	0.393
MAP3K7	0.168	0.192	0.394
CCL21	-0.816	0.936	0.395
HLA-A	0.669	0.781	0.403
IL10	0.742	0.867	0.405
EZH2	-0.258	0.306	0.411
BATF3	-0.354	0.42	0.411
IL15	0.467	0.554	0.412
TNFSF10	0.363	0.432	0.412
PRR5	-0.364	0.437	0.416

PARP12	0.303	0.364	0.416
TAPBP	0.18	0.218	0.419
PVR	0.443	0.536	0.420
BAD	0.286	0.348	0.424
CEACAM3	0.783	0.954	0.424
TNFAIP6	0.624	0.765	0.426
CXCL3	0.711	0.881	0.431
FAM124B	0.556	0.693	0.433
TNFSF18	-0.804	1	0.435
MFNG	-0.462	0.58	0.436
PIK3CG	0.262	0.329	0.438
JAG1	0.402	0.506	0.438
SHC2	-0.593	0.75	0.440
APC	0.213	0.271	0.443
CTSW	0.548	0.701	0.446
ADM	0.466	0.597	0.446
PTGS2	-0.711	0.916	0.448
SMAP1	0.133	0.172	0.451
TPI1	0.258	0.334	0.451
FLT1	0.428	0.555	0.451
WNT5B	-0.591	0.765	0.451
NLRP3	-0.622	0.81	0.453
ALDOA	0.338	0.441	0.454
FYN	-0.144	0.189	0.456
COL6A3	0.48	0.63	0.456
CCL19	-0.527	0.697	0.460
IRF4	0.533	0.707	0.461
ANGPTL4	0.553	0.735	0.462
ICAM5	-0.614	0.817	0.463
ITGB8	-0.399	0.533	0.464
NFKBIE	0.295	0.397	0.468
HLA-E	0.247	0.335	0.471
GIMAP6	-0.29	0.396	0.474
CCNO	-0.736	1.01	0.476
HLA-C	-0.28	0.386	0.478
LRRC32	-0.232	0.32	0.478
MS4A6A	0.328	0.453	0.479
IL12RB2	-0.471	0.651	0.479
HK1	0.183	0.254	0.480
ICOS	0.688	0.979	0.492
GIMAP4	0.304	0.433	0.493
MAGEA3/A6	1.05	1.5	0.495
HLA-DQA2	-0.977	1.41	0.497
ARID1A	0.63	0.923	0.505
HCK	0.267	0.393	0.506
TNFRSF10B	0.325	0.48	0.508
BNIP3	0.289	0.431	0.512
CCL13	0.496	0.741	0.512
JAG2	-0.45	0.675	0.514
THBD	0.381	0.573	0.515
CEBPB	0.264	0.399	0.517
SPRY4	-0.315	0.479	0.519
SNCA	0.325	0.494	0.520
MMP1	0.895	1.36	0.520
FPR1	0.434	0.667	0.523
WNT10A	0.462	0.709	0.524
TNFRSF1B	0.383	0.594	0.528
CXCR2	-0.545	0.85	0.530
ARG2	0.353	0.564	0.540
PIK3R1	0.247	0.397	0.541
VEGFA	0.375	0.603	0.542
INHBA	-0.538	0.867	0.543
TGFB2	0.419	0.679	0.545
MAP3K12	0.68	1.1	0.546
GMIP	0.762	1.24	0.547
TLR8	-0.363	0.596	0.551
HIF1A	0.366	0.608	0.556
S100A8	0.924	1.54	0.556
LILRA5	-0.475	0.795	0.558
SFRP1	-0.42	0.705	0.559
RAD51C	-0.27	0.454	0.560

SERPINB5	-1.18	1.99	0.561
PIAS4	0.199	0.34	0.566
VCAN	0.304	0.519	0.566
MAP3K8	0.25	0.428	0.566
CD58	0.257	0.44	0.567
HRAS	0.372	0.639	0.568
SIRPA	0.291	0.502	0.570
GOT2	0.16	0.277	0.570
FANCA	-0.31	0.535	0.570
TPSAB1/B2	-0.33	0.571	0.571
NFATC2	0.164	0.285	0.572
GOT1	-0.255	0.445	0.575
ARNT2	-0.455	0.796	0.575
NCR1	0.461	0.817	0.581
CD40LG	0.374	0.664	0.581
IL18R1	0.684	1.23	0.585
ITGA1	0.29	0.525	0.587
PPARG	-0.284	0.52	0.592
KIT	-0.314	0.578	0.593
TGFB3	0.319	0.587	0.594
CTNNB1	0.138	0.257	0.598
TCF3	0.128	0.239	0.599
PGPEP1	0.273	0.512	0.601
TNFSF13B	-0.323	0.609	0.602
ANGPT2	-0.228	0.43	0.603
HDAC11	-0.155	0.294	0.604
DNMT1	-0.236	0.447	0.604
MS4A2	0.408	0.784	0.610
TNFRSF10D	0.285	0.552	0.611
ICAM2	0.266	0.519	0.614
TLR5	-0.457	0.89	0.615
SFXN1	-0.178	0.348	0.615
WDR76	-0.652	1.27	0.616
CASP9	0.163	0.32	0.617
CCND1	-0.246	0.483	0.617
FOXP3	-0.513	1.01	0.618
FAS	0.158	0.312	0.619
COL17A1	-0.887	1.76	0.621
ENTPD1	0.176	0.35	0.622
CD274	0.26	0.518	0.622
CCR4	-0.325	0.653	0.625
IRF8	0.248	0.501	0.628
PMS2	-0.111	0.225	0.629
BNIP3L	0.289	0.589	0.630
GZMM	0.361	0.738	0.632
PALMD	0.214	0.442	0.634
A2M	0.234	0.493	0.641
SLC2A1	0.211	0.446	0.642
FGFR1	0.149	0.32	0.649
MYCT1	0.286	0.618	0.649
VTCN1	-0.325	0.708	0.653
NLRC5	-0.204	0.446	0.653
PDGFRB	-0.262	0.575	0.654
PTPN11	0.0984	0.219	0.658
TLR7	-0.459	1.04	0.665
TMEM140	-0.138	0.316	0.667
PRKAA2	-0.305	0.699	0.668
MXI1	0.117	0.269	0.669
IDO1	-0.309	0.715	0.671
NECTIN1	0.239	0.562	0.676
LTB	0.294	0.692	0.676
SELP	0.474	1.12	0.676
C5AR1	-0.321	0.757	0.677
RPL23	0.146	0.345	0.678
TICAM1	0.108	0.256	0.679
NOD2	0.156	0.376	0.683
EGFR	0.203	0.49	0.684
TBX21	0.328	0.793	0.684
CST2	0.631	1.53	0.685
MAPK10	0.336	0.827	0.690
MLH1	-0.124	0.309	0.692

CXCR3	-0.341	0.846	0.692
F2RL1	-0.233	0.597	0.701
PDGFA	0.148	0.381	0.702
PECAM1	0.152	0.392	0.704
CPA3	-0.308	0.8	0.705
SELL	0.225	0.591	0.708
IFITM2	0.167	0.441	0.710
BIRC5	0.157	0.423	0.715
IL1R2	0.259	0.699	0.716
CD209	-0.155	0.421	0.717
BRD4	0.118	0.322	0.718
BMP2	0.212	0.579	0.719
CD80	0.166	0.46	0.723
HDAC4	0.142	0.394	0.723
ESR1	-0.299	0.832	0.724
EOMES	0.433	1.23	0.729
BID	0.112	0.318	0.730
NID2	-0.21	0.604	0.732
ANGPT1	0.234	0.673	0.733
THY1	-0.221	0.64	0.734
LY9	0.205	0.593	0.734
C5	0.17	0.493	0.734
RRM2	-0.139	0.405	0.735
FGF13	0.222	0.667	0.743
SLC7A5	-0.131	0.399	0.747
HDAC5	0.163	0.498	0.748
CD3G	0.3	0.92	0.748
CXCL12	-0.205	0.634	0.750
CXCL9	0.404	1.28	0.756
LOXL2	0.138	0.44	0.757
FBP1	0.207	0.659	0.757
PSMB5	0.0737	0.237	0.759
BRCA1	0.125	0.404	0.760
MSH2	-0.0949	0.308	0.762
IER3	0.207	0.677	0.763
SLC1A5	0.0804	0.263	0.764
BRCA2	-0.104	0.342	0.764
SERPINH1	-0.153	0.504	0.765
GPC4	0.187	0.633	0.771
CCNB1	-0.119	0.404	0.771
SOCS1	0.176	0.597	0.772
VSIR	-0.158	0.541	0.773
MICA	-0.126	0.431	0.774
LYZ	0.158	0.547	0.777
SOX10	-0.205	0.719	0.780
HLA-DRB1	0.115	0.406	0.781
DUSP5	0.1	0.363	0.786
NFIL3	-0.119	0.432	0.786
SMAD5	0.124	0.451	0.787
CCNA1	-0.193	0.712	0.790
CSF2RB	-0.125	0.465	0.792
MGMT	0.0807	0.302	0.793
PFKM	-0.0817	0.311	0.796
TAP1	0.133	0.508	0.797
ERBB2	0.143	0.566	0.803
NGFR	-0.148	0.585	0.803
UBE2C	-0.122	0.489	0.806
TAP2	0.0651	0.263	0.808
CCL14	-0.186	0.76	0.810
GZMH	0.267	1.12	0.814
COL4A5	0.118	0.493	0.814
CD79B	0.254	1.07	0.814
CEP55	-0.126	0.536	0.816
RPS6KB1	-0.0558	0.238	0.818
TNFRSF18	0.25	1.07	0.818
BAMBI	0.156	0.675	0.819
CDH5	-0.0975	0.436	0.826
CD36	0.108	0.485	0.826
GLS	-0.143	0.657	0.830
ADGRE1	0.181	0.841	0.832
BBS1	-0.0863	0.412	0.837

CD40	0.101	0.482	0.837
BLM	-0.145	0.71	0.841
FZD8	-0.117	0.58	0.842
DLL1	-0.092	0.457	0.843
STAT3	0.0803	0.404	0.845
CCNE1	-0.0775	0.399	0.848
CDKN1C	-0.0856	0.443	0.849
TGFBR2	0.12	0.627	0.850
NFKBIA	0.114	0.628	0.858
FCN1	-0.0886	0.49	0.859
JAK1	-0.0574	0.321	0.860
TNFSF4	0.158	0.888	0.861
SIRPB2	0.159	0.899	0.862
CX3CL1	0.0828	0.476	0.864
TNFSF8	-0.153	0.904	0.868
CENPF	-0.0865	0.528	0.872
SELE	-0.11	0.676	0.873
GHR	-0.0832	0.517	0.874
HES1	0.059	0.369	0.875
WNT11	-0.0905	0.565	0.875
IKBKG	0.0716	0.464	0.879
ITGAE	0.0384	0.252	0.881
ALDOC	0.063	0.414	0.881
BRIP1	0.0513	0.341	0.882
TNF	0.0644	0.439	0.885
SLC11A1	-0.132	0.916	0.887
CBLC	0.0959	0.698	0.892
TLR9	-0.106	0.78	0.894
HDC	0.0695	0.525	0.896
TNFRSF10C	0.0893	0.711	0.901
HLA-DOB	0.0871	0.717	0.905
LILRB2	0.0454	0.38	0.906
BTLA	0.0794	0.69	0.910
PDGFB	0.0354	0.309	0.910
CSF3R	-0.0947	0.904	0.918
PIK3R2	0.0508	0.496	0.920
CDKN2B	0.0589	0.578	0.920
IL18	0.0812	0.817	0.922
HEY1	0.045	0.502	0.930
MSH6	-0.0386	0.438	0.931
EPCAM	-0.0599	0.732	0.936
RAD51	0.0789	1.12	0.945
IL11RA	0.0341	0.512	0.948
CXCL6	0.096	1.66	0.954
EGF	-0.0405	0.721	0.956
WNT4	-0.0371	0.707	0.959
CD8B	0.0415	0.805	0.960
NECTIN2	0.0168	0.328	0.960
ICOSLG	0.0139	0.354	0.969
KIF2C	-0.0162	0.427	0.970
GAS1	0.0157	0.531	0.977
PC	0.0123	0.423	0.977
CLECL1	-0.0197	0.793	0.980
TNFRSF25	0.0103	0.563	0.986
ITPK1	0.00312	0.226	0.989
SNAI1	-0.00374	0.683	0.996
RPTOR	0.000399	0.45	0.999
BRD3	-0.00022	0.392	1.000

Supplementary Table 12. Correlation of NanoString pathway scores with chemotherapy response

Pathway	Scores (median)		p-value
	Responders	Non-responders	
Angiogenesis	-0.568	1.448	0.026
Matrix Remodeling and Metastasis	-0.850	2.705	0.020
Cytokine and Chemokine Signaling	-1.438	3.445	0.005
Myeloid Compartment	-0.946	3.651	0.007
Hypoxia	0.401	1.267	0.152
Antigen Presentation	-1.783	1.119	0.238
Cytotoxicity	-1.128	1.492	0.091
Interferon Signaling	-0.730	2.831	0.075
Costimulatory Signaling	-2.018	1.593	0.051
Lymphoid Compartment	-0.496	1.329	0.129
NF-kappaB Signaling	-0.832	0.677	0.600
Immune Cell Adhesion and Migration	-0.398	2.372	0.033
JAK-STAT Signaling	0.089	1.971	0.004
Apoptosis	0.606	-0.154	0.778
Metabolic Stress	0.465	0.861	0.395
Notch Signaling	-0.207	-0.996	0.272
MAPK	-1.321	0.163	0.904
PI3K-Akt	-1.451	0.049	0.840
Wnt Signaling	-0.184	0.011	0.717
Autophagy	-0.694	-0.273	0.600
Cell Proliferation	-0.379	-0.114	0.904
DNA Damage Repair	-0.690	-0.805	0.904
Epigenetic Regulation	-0.795	-0.037	0.904
Hedgehog Signaling	0.245	0.023	0.492
TGF-beta Signaling	0.619	0.693	0.395
